# Dihuang-Yinzi Alleviates Cognition Deficits *via* Targeting Energy-Related Metabolism in an Alzheimer Mouse Model as Demonstrated by Integration of Metabolomics and Network Pharmacology

**DOI:** 10.3389/fnagi.2022.873929

**Published:** 2022-04-01

**Authors:** Guanghui Han, Weizhe Zhen, Yuan Dai, Hongni Yu, Dongyue Li, Tao Ma

**Affiliations:** ^1^Dongfang Hospital, Beijing University of Chinese Medicine, Beijing, China; ^2^School of Health Preservation and Rehabilitation, Chengdu University of Traditional Chinese Medicine, Chengdu, China; ^3^College of Traditional Chinese Medicine, Beijing University of Chinese Medicine, Beijing, China

**Keywords:** Alzheimer’s disease, Dihuang-Yinzi, metabolomics, network pharmacology, energy metabolism, mitochondria, reactive oxygen species

## Abstract

Energy metabolism disturbance and the consequent reactive oxygen species (ROS) overproduction play a key and pathogenic role in the onset and progression of Alzheimer’s disease (AD). Dihuang-Yinzi (DHYZ) is a traditional Chinese herbal prescription clinically applied to treat AD and other neurodegenerative diseases for a long time. However, the systematical metabolic mechanism of DHYZ against AD remains largely unclear. Here we aimed to explore the mechanism of DHYZ in the treatment of AD comprehensively in an *in vivo* metabolic context by performing metabolomics analysis coupled with network pharmacology study and experimental validation. The network pharmacology was applied to dig out the potential target of DHYZ against AD. The metabolomics analysis based on UPLC-HRMS was carried out to profile the urine of 2× Tg-AD mice treated with DHYZ. By integrating network pharmacology and metabolomics, we found DHYZ could ameliorate 4 key energy-related metabolic pathways, including glycerophospholipid metabolism, nicotinate/nicotinamide metabolism, glycolysis, and tricarboxylic acid cycle. Besides, we identified 5 potential anti-AD targets of DHYZ, including DAO, HIF1A, PARP1, ALDH3B2, and ACHE, and 14 key differential metabolites involved in the 4 key energy-related metabolic pathways. Furthermore, DHYZ depressed the mitochondrial dysfunction and the resultant ROS overproduction through ameliorating glycerophospholipid metabolism disturbance. Thereby DHYZ increased nicotinamide adenine dinucleotide (NAD^+^) content and promoted glycolysis and tricarboxylic acid (TCA) cycle, and consequently improved oxidative phosphorylation and energy metabolism. In the present study, we provided a novel, comprehensive and systematic insight into investigating the therapeutic efficacy of DHYZ against AD *via* ameliorating energy-related metabolism.

## Introduction

Alzheimer’s disease (AD) is complex neurodegenerative dementia in aging people and places a substantial burden on the economy and psychology on society (Grubman et al., 2019). With the in-depth study of AD, the abnormality of energy metabolism has attracted a lot of attention in recent years (Minhas et al., 2021). As an organ with the highest energy requirements, the brain is vulnerable to energy metabolism impairments. The energy metabolism is composed of several important metabolic pathways, including glycolysis, tricarboxylic acid (TCA) cycle, the pentose phosphate pathway (PPP), oxidative phosphorylation (OxPhos), etc. It has shown that the astrocyte glycolysis is disturbed resulting in a decrease in glucose utilization, and deficits in synaptic plasticity and memory of AD patients and mouse models (Le Douce et al., 2020). The abnormalities in the TCA cycle and OxPhos are also closely related to AD pathology (Shaerzadeh et al., 2014; Pawlosky et al., 2017; Puchowicz and Seyfried, 2017). β-amyloid (Aβ), one of the most important hallmarks of AD, can cause the chronic reprogramming of energy-related metabolisms, including OxPhos and glycolysis (Baik et al., 2019).

Metabolomics is a powerful phenotyping technique that identifies and quantifies small molecular metabolites and their complements in cells, tissues, and biological fluids, and provides a highly sensitive and specific method for measurement of multi-parameters of disease phenotypes at a systematic level (van der Velpen et al., 2019). The applications of metabolomics in AD help us to identify the changes of metabolites in the brain, serum, and urine, which may play an important role in the pathogenesis of AD at preclinical and clinical stages (Woo et al., 2010; Liu et al., 2013; Mapstone et al., 2014; Shao et al., 2020). Given the fact that the metabolic variation is reversible in the early stage of AD, profiling the metabolomic characteristics of the pathological progression of AD is of particular relevance for the treatment or impeding disease progression (Oresic et al., 2011; Mapstone et al., 2014). In the recent decade, many metabolomics studies had been performed to discover and identify differential metabolites or biomarkers to facilitate understanding of the pathogenesis, diagnosis, and prognosis of AD, and evaluating of the therapeutic efficacy of drugs against AD by using plasma, serum, cerebrospinal fluid, saliva, and urine samples (Oresic et al., 2011; Tsuruoka et al., 2013; Mapstone et al., 2014). Since urinary metabolomics analysis has many advantages, such as non-invasive, sensitive, convenient, rapid, etc., it has received great attention in AD treatment, diagnosis, and drug evaluation (Liu et al., 2019; Wei et al., 2019; Wang et al., 2021).

Network pharmacology is a comprehensive technique integrating traditional pharmacology, bioinformatics, chemoinformatics, and systems biology. The application of network pharmacology provides us with the objective evidence of the herbal prescription intervention in AD and sheds light on developing novel drugs for AD treatment (Zeng et al., 2019; Gu et al., 2020). Given network pharmacology is an effective way to predict the complex mechanisms of traditional Chinese herbal prescriptions, it has widely been used in clarifying their multi-target effect on neurodegenerative diseases, including in AD, combing with metabolomics, or other omics (Yuan et al., 2017; Yun et al., 2021).

Dihuang-Yinzi (DHYZ) is a traditional Chinese herbal prescription, mainly composed of 15 herbs, such as Rehmannia, Cistanche, Dogwood, Morinda, etc. (Table 1). Clinically, DHYZ has been used in the treatment of neurodegenerative diseases, including AD, for a long time (Lee et al., 2021). In recent years, many studies demonstrated that DHYZ exhibits neuroprotective functions in a variety of neurological diseases (Hu et al., 2009; Qiu et al., 2016). DHYZ can inhibit neuronal apoptosis through mitochondria- and endoplasmic reticulum-dependent pathways, induce expression of glial-derived neurotrophic factor (GDNF), and maintain the ultrastructure of the blood-brain barrier (BBB) (Zhang et al., 2017; Zhang J. et al., 2018). Furthermore, DHYZ improved the cognition of ischemic mice by depressing the expression of extracellular signal-regulated protein kinase (ERK) and synaptophysin (SYP) (Hu et al., 2009). A study based on a meta-analysis shows that DHYZ can significantly improve the cognitive function and daily activities of AD patients (Lee et al., 2021). Our previous studies have shown that DHYZ can ameliorate the learning and memory impairments of rat and mouse model of AD by depressing mitochondrial impairments and improving energy production (Ma et al., 2012; Huang et al., 2018; Yan et al., 2018). However, the comprehensive mechanism of therapeutic effects of DHYZ against AD especially at holistic metabolic level remains elusive so far.

**TABLE 1:** Component herbs of DHYZ.

Pharmaceutical name	Botanical plant name	Family and plant parts used	English name	Chinese name	Amount in preparation (g)
Cistanches Herba	*Cistanche deserticola* Y.C.Ma	Orobanchaceae; Fleshy stem	cistanche	Rou Cong Rong	15
Morindae Officinalis Radix	*Morinda officinalis* How	Rubiaceae; Root	morinda	Ba Ji Tian	15
Rehmanniae Radix Praeparata	*Rehmannia glutinosa* Libosch	Scrophulariaceae; Root tuber	rehmannia	Shu Di Hang	15
Corni Fructus	*Cornus officinalis* Sieb. et Zucc	Cornaceae; fruit	dogwood	Shan Zhu Yu	15
Aconiti Lateralis Radix Praeparata	*Aconitum carmichaelii* Debx.	Ranunculaceae; Subroot	aconite	Zhi Fu Zi	15
Cinnamomi Cortex	*Cinnamomum cassia* Presl	Lauraceae; bark	cinnamon	Rou Gui	15
Ophiopogonis Radix	*Ophiopogon japonicus* (L.f) Ker-Gawl.	Liliaceae; Root tuber	ophiopogon	Mai Dong	15
Dendrobii Caulis	*Dendrobium nobile* Lindl.	Orchidaceae; stem	dendrobium	Shi Hu	15
Schisandrae Chinensis Fructus	*Schisandra chinensis* (Turcz.) Baill	Magnoliaceae; fruit	schisandrae	Wu Wei Zi	15
Polygalae Radix	*Polygala tenuifolia* Willd.	Polygalaceae; Root	polygala	Yuan Zhi	15
Acori Tatarinowii Rhizoma	*Acorus tatarinowii* Schott	Araceae; tuber	calamus	Shi Chang Pu	15
Poria	*Poria cocos* (Schw.) Wolf	Polypores; sclerotium	poria	Fu Ling	15
Menthae Haplocalycis Herba	*Mentha haplocalyx* Briq.	Labiatae; Aerial part	mint	Bo He	15
Zingiberis Rhizoma Recens	*Zingiber officinale* Rosc.	Zingiberaceae; rhizome	ginger	Sheng Jiang	10
Jujubae Fructus	*Ziziphus jujuba* Mill.	Jujubae; fruit	jujube	Da Zao	5

**FIGURE 1:**
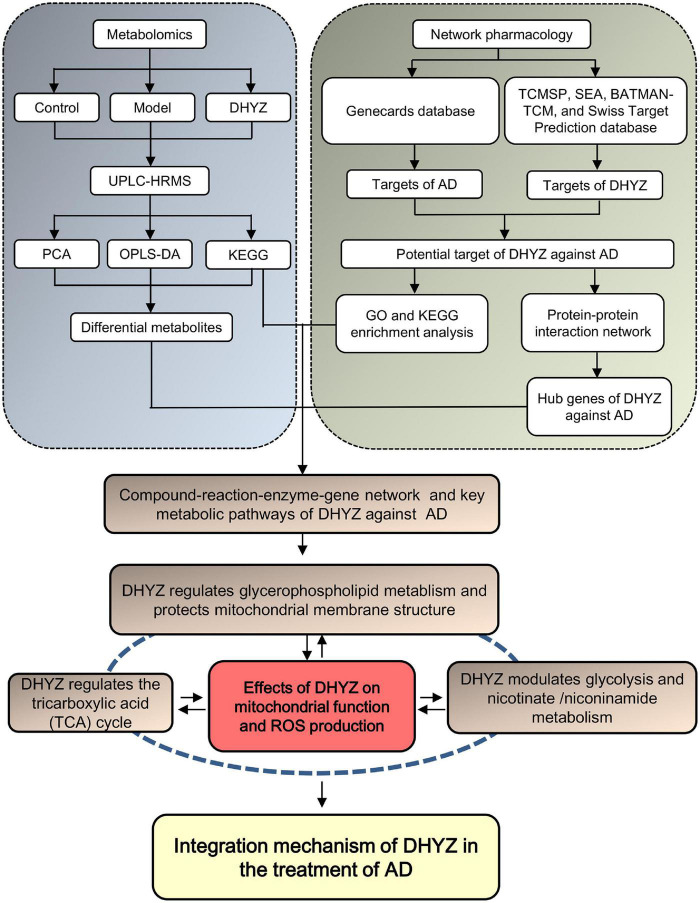
Schematic diagram of the integrated study of DHYZ against AD combining urine metabonomics and network pharmacology.

In the present study, we performed metabolomics analysis coupled with a network pharmacology study to explore the key metabolites and their related metabolic pathways in treatment with DHYZ, and key targets of DHYZ against AD (Figure 1). Our result provided a novel multi-dimensional perspective of the therapeutic efficacy of DHYZ against AD and a scientific basis for its further clinical precision medication.

## Materials and Methods

### Herbal Materials

The herbal materials, namely Cistanches Herba (*Cistanche deserticola* Y.C.Ma), Morindae Officinalis Radix (*Morinda officinalis* How), Rehmanniae Radix Praeparata (*Rehmannia glutinosa* Libosch), Corni Fructus (*Cornus officinalis* Sieb. et Zucc), Aconiti Lateralis Radix Praeparata (*Aconitum carmichaelii* Debx.), Cinnamomi Cortex (*Cinnamomum cassia* Presl), Ophiopogonis Radix (*Ophiopogon japonicus* (L.f) Ker-Gawl.), Dendrobii Caulis (*Dendrobium nobile* Lindl.), Schisandrae Chinensis Fructus (*Schisandra chinensis* (Turcz.) Baill.), Polygalae Radix (*Polygala tenuifolia* Willd.), Acori Tatarinowii Rhizoma (*Acorus tatarinowii* Schott), Poria (*Poria cocos* (Schw.) Wolf), Menthae Haplocalycis Herba (*Mentha haplocalyx* Briq.), Zingiberis Rhizoma Recens (*Zingiber officinale* Rosc.), and Jujubae Fructus (*Ziziphus jujuba* Mill.) used for the preparation of DHYZ were obtained from Dongfang Hospital, Beijing University of Chinese Medicine. All the above-mentioned herbal medicines used to prepare DHYZ have been kindly authenticated by Dr. Yuan Dai (Associate professor, Chengdu University of Traditional Chinese Medicine).

### Preparation of Dihuang-Yinzi Extract

The formula of DHYZ (one dose) is presented in Table 1 as described previously (Huang et al., 2018). All the herbs in DHYZ (10 doses) were added in distilled water (1:4 w/v), soaked for 2 h, and extracted at 100°C for 2 times, 2 h each time. The decoctions were mixed, filtered, and dried by decompressing at 60°C to the drying extract (5.53 g of herbal mixtures generated 1 g drying extract). Then the drying powder was mixed thoroughly, stored at 4°C, and dissolved in normal saline at the desired concentrations before use.

### Reagents and Materials

Standards of loganin (Lot No. 36483), echinacoside (Lot No. 07538), Verbascoside (Lot No. V4015), Erianin (Lot No. PHL83499), Cinnamic Acid (Lot No. C80857), Tenuifolin (Lot No. PHL83546), Quercetin (Lot No. PHR1488), Ruscogenin (Lot No. SMB00295), Kaempferol (Lot No. 60010), Beta-sitosterol (Lot No. S1270), and Schisandrin (Lot No. SML0054) were purchased from Sigma-Aldrich Co. LLC (St. Louis, MO, United States). Formic acid, methanol, ammonium acetate, ammonium hydroxide, and acetonitrile used in UPLC were provided by Fisher Scientific Co. LLC (Fair Lawn, NJ, United States). The ultrapure water (18.2 MΩ⋅cm) used in the experiment was prepared with Milli-Q IQ 7003 purified water system (Merck Millipore, Darmstadt, Germany).

### Fingerprint Spectrum Analysis

The fingerprint spectrum of multi-components of the DHYZ was characterized by ultrahigh-pressure liquid chromatography (UPLC). The analyses were performed with Waters ACQUITY UPLC system (Waters Corp., Milford, MA, United States) equipped with a photo-diode array (PDA), a quaternary pump, an autosampler, and empower software. Chromatographic separation was performed on the Waters BEH C_18_ column (50 mm × 2.1 mm, 1.7 μm) at 40°C with the detector of wavelength 220 nm. The mobile phase was a mixture of water containing 0.1% formic acid (A) and acetonitrile containing 0.1% formic acid (B). A linear gradient elution was conducted as follows: 0–3 min, 5–10%B; 3–10 min, 10–85%B; 10–15 min, 95%B; 15–20 min, 5%B. The flow rate was 0.4 mL/min and the total injection volume was 2 μL.

### Animals and Grouping

Three-month-old male APPswe/PS1dE9 (2× Tg-AD) mice which harbor human APPswe (Swedish mutations K594N/M595L) and presenilin-1 with exon 9 deleted (PS1dE9) under the control of the constitutively active PrP gene promoter (Jankowsky et al., 2004) and non-transgenic (Non-Tg) littermates of the same month were provided by the Nanjing Biomedical Research Institute of Nanjing University (Nanjing, China). All mice were adaptively reared for 30 days in the SPF animal laboratory in Dongfang Hospital, Beijing University of Chinese Medicine, China. After adaptive breeding, 4-month-old Non-Tg mice (*n* = 20) were designed as the control group, and 2× Tg-AD mice were randomly divided into model group and DHYZ group, with 20 mice in each group. All the mice are housed individually in cages, the ambient temperature is 23 ± 1°C, and relative humidity is 55 ± 5% under. All mice were raised in an environment with a cycle of 12h:12h light/dark, with the intake of food and water *ad libitum*. All the animals were handled according to the NIH Guide for the Care and Use of Laboratory Animals (NIH Publications No. 80-23, revised 1996). All animal studies were approved by the Animal Care and Welfare Committee of Dongfang Hospital, Beijing University of Chinese Medicine, China.

### Administration of Dihuang-Yinzi

Estimate the dosage of DHYZ in mice based on the dosage for adults (crude drug: 3.5 g/kg BW•day). All 4-month old mice were intragastrically administered DHYZ at a dose of 35 g crude drug/kg BW•day (6.3 g drying extract/kg BW•day) for 180 days, once a day. The mice in the control group and the model group were intragastrically given the same volume of sterilized saline for 180 days, once a day.

### Morris Water Maze

patial learning and memory were analyzed using the Morris water maze (MWM) tests which were performed as described previously (Dai et al., 2020). Briefly, the place navigation test included 5 days of training, 2 pieces of training a day, and the interval between the 2 pieces of training was more than 1 h. 24 h after the end of the place navigation test, that is, the 6th day of the experiment, the spatial probe test was performed. From the 7th to the 9th day of the experiment, the visible-platform test was performed to evaluate the impact of mouse vision on the ability to search the platform.

### Network Pharmacology Analysis

The composite ingredients of Cinnamon, Dendrobium, Ophiopogon, and Polygala were searched in Bioinformatics Analysis Tool for Molecular Mechanism of Traditional Chinese Medicine (BATMAN-TCM^1^), and screened with the high gastrointestinal absorption (GI absorption) and drug-like properties (DL) in SwissADME^2^. The composite ingredients of other herbs in DHYZ were obtained by searching in the Traditional Chinese Medicine Systems Pharmacology Database and Analysis Platform (TCMSP^3^). The composite ingredients were filtered with oral bioavailability (OB) ≥30%, and drug-like property (DL) ≥0.18. The chemical structure and SMILES number of all composite ingredients were obtained with the PubChem^4^. The molecular targets of these composite ingredients were screened by importing the chemical structure formula into the SwissTargetPrediction^5^ database and the SMILES number into the SEA^6^ database. The candidate therapeutic targets of AD were collected by searching the keywords of “Alzheimer” in the genecards^7^ database. The intersection of molecular targets of DHYZ and candidate therapeutic targets for AD treatment is considered to be the predicted targets for DHYZ against AD. All the predicted targets were imported into UniProtKB^8^ to standardize the gene and protein names. The predicted targets were imported into the Metascape^9^ for GO and Kyoto Encyclopedia of Genes and Genomes (KEGG) enrichment analysis, with the KEGG pathway analysis was set as *P* < 0.05. The herbs-composite ingredients-potential targets network was established by utilizing the Cytoscape 3.8.2 (National Institute of General Medical Science, United States). The predicted targets of DHYZ for AD treatment were imported into STRING 11.0^10^ to construct the PPI network, and the hub genes were screened by CytoHubba in Cytoscape 3.8.2.

### Metabolomics Analysis

#### Sample Preparation

After intragastrically administrated for 180 days, the mice were put in 3600M021 Mice Metabolic Cages (TECNIPLAST S.p. A, BUGUGGIATE, VA, Italy), and the urine samples were collected and centrifuged. All urine samples were stored at −80°C before UPLC-HRMS Analysis.

#### Untargeted Ultra-High-Performance Liquid Chromatography-High-Resolution Mass Spectrometer Analysis

Separation conditions by UPLC Untargeted metabolomics analysis were conducted by using 3 different analytical methods on an Ultimate 3000 ultra-high-performance liquid chromatography coupled with Q Exactive™ quadrupole-Orbitrap high-resolution mass spectrometer UPLC-HRMS system (Thermo Fisher Scientific, United States). For methods 1 and 2 (M1, 2), the polar metabolome extracts were profiled on reverse-phase chromatographic separation with positive and negative ionization detection, respectively. Metabolites were separated by using an Acquity™ HSS C18 column (Waters Co., United States, 2.1 mm × 100 mm) for M1. Furthermore, other mobile phases were employed to eluted metabolites separated on an Acquity™ BEH C18 column (Waters Co., United States, 1.7 μm, 2.1 mm × 100 mm). As for Method 3, HILIC separation was utilized by using an Acquity™ BEH amide column (Waters Co., United States, 1.7 μm, 2.1 mm × 100 mm) after 5 μL aliquots of metabolites extract injected, and 5% water in acetonitrile as weak eluent and 40% acetonitrile in water as strong eluent which was both added ammonium acetate to as buffer salt to improve separation.

#### Data Processing and Analysis

The full scan and data-dependent MS2 metabolic profiles data were further processed with Compound Discoverer (CD version 3.0 Thermo Fisher Scientific) for comprehensive component extraction. The polar metabolites were structurally annotated through searching acquired MS2 against a local proprietary iPhenome™ SMOL high-resolution MS/MS spectrum library created using authentic standards, NIST 17 Tandem MS/MS library (National Institute of Standards and Technology), local version MoNA (MassBank of North America), as well as mzCloud library (Thermo Fisher Scientific, United States). Besides, the exact m/z of MS1 spectra was searched against a local KEGG, HMDB metabolite chemical database. For metabolite identification or structural annotation, mass accuracy of precursor within ±5ppm was a prerequisite, meanwhile, isotopic information including at least 1 isotopes within 10 ppm and a fit score of relative isotopic abundance pattern 70% were introduced to confirm the chemical formula in addition to exact mass. Furthermore, retention time information as well as high-resolution MS/MS spectra similarity was employed to strictly confirm the structural annotation of metabolites. The area under curve values as extracted as quantitative information of metabolites with XCalibur Quan Browser information, all peak areas data for the annotated metabolites were exported into Excel software for trim and organization before statistics (Microsoft, United States).

The metabolome data deriving from different measurements were merged and those detected with multiple methods were excluded to guarantee uniqueness of metabolite and lipid, and then Log2 was transformed for final statistical analysis. The principal component analysis (PCA), as well as the orthogonal partial least square-discriminant analysis (OPLS-DA), was conducted with SIMCA-P software (Umetrics, Sweden), and another univariate analysis including independent sample *t*-test and *p*-value FDR adjust, as well as metabolic pathway analysis was conduct on the MetaboAnalyst website.

#### Kyoto Encyclopedia of Genes and Genomes Enrichment Analysis of Differential Metabolites

The intersection of the differential metabolites of the model group *vs.* the control group and the DHYZ group *vs.* model group are considered to be the main differential metabolites involved in the AD treatment by DHYZ. All these metabolites were inputted in MetaboAnalyst 5.0^11^ for KEGG enrichment and pathway analysis.

### Compound-Reaction-Enzyme-Gene Network Construction

The differential metabolites and hub genes closely related to DHYZ treatment of AD were imported into MetScape in Cytoscape 3.8.2 to construct a visual network including the interactions among metabolites, hub genes, pathways, and enzymes.

### Antibodies and Western Blot Analysis

Primary antibodies used in Western blot analysis were as follows: polyclonal anti D-amino-acid oxidase (DAO) antibody (1:1000, ab187525, abcam, Cambridge, United Kingdom), polyclonal anti- aldehyde dehydrogenase 3 family member B2 (ALDH3B2) antibody (1:1000, SAB1410380, Sigma-Aldrich, Merck KGaA Co, Darmstadt, Germany), monoclonal anti-hypoxia-inducible factor 1-alpha (HIF1A) antibody (1:1000, #36169, Cell Signaling Technology, MA, United States), monoclonal anti-Poly [ADP-ribose] polymerase 1 (PARP-1) antibody (1:1000, #9532S, Cell Signaling Technology, MA, United States), monoclonal anti-acetylcholinesterase (AChE) antibody (1:500, sc-373901, Santa Cruz Biotech., Inc., TX, United States), monoclonal anti-pyruvate dehydrogenase kinase 1 (PDHK1) antibody (1:1000, #3820, Cell Signaling Technology, MA, United States), monoclonal anti-pyruvate dehydrogenase (PDH) antibody (1:1000, #3205, Cell Signaling Technology, MA, United States), polyclonal anti-phospho-pyruvate dehydrogenase (phospho-PDH) antibody (1:1000, #31866, Cell Signaling Technology, MA, United States), monoclonal anti-β actin antibody (C4) (1:2000, sc-47778, Santa Cruz Biotech., Inc., TX, United States), monoclonal anti-GAPDH antibody (1:2000, sc-47724, Santa Cruz Biotech., Inc., TX, United States). Goat anti-rabbit IgG-HRP (1:5000, sc-2004) and goat anti-mouse IgG-HRP (1:5000, sc-2302) secondary antibody were provided by Santa Cruz Biotech., Inc. (TX, United States).

After being dissected, the mouse brains were homogenized in ice-cold RIPA lysis buffer (Applygen Technologies, Beijing, China) supplemented with cOmplete protease inhibitor cocktail (Roche Diagnostic GmbH, Mannheim, Germany) with Dounce homogenizer. The brain homogenates were centrifuged at 12,000 *g* and 4°C for 10 min, and the supernatant was transferred to a new EP tube for later assay. After protein quantification using the Pierce BCA protein assay kit (Thermo Fisher Scientific, IL, United States), 50 μg of total protein lysate was subjected to SDS-PAGE and electrophoretically transferred to polyvinylidene fluoride (PVDF) membranes (Millipore, MA, United States). Primary antibodies were used to detect the corresponding protein level in the mouse brain. GAPDH and β-actin served as loading control of total protein. Immunoreactive bands were detected with species-specific HRP-conjugated secondary antibodies, developed with Pierce ECL Western Blotting Substrate (Thermo Fisher Scientific, IL, United States), and images were captured with GeneGnome XRQ bio imaging system (Syngene Inc., Cambridge, United Kingdom). The images were analyzed and quantified with the Image J 1.46r (NIH, United States).

### Energy Charge Measurement

After being deeply anesthetized by using sodium pentobarbital, mouse brains were excised. 5 mouse brains from each group were homogenized with cold saline solution, added to 4% perchloric acid to precipitate the protein, and centrifuged at 3000 rpm for 10 min at 4°C. The supernatant was neutralized with potassium hydroxide and subjected to reverse-phase HPLC (Microsorb C18; Rainin Instruments Co. Swiss). The column eluate was monitored at an absorbance of 254 nm, and the adenosine triphosphate (ATP), adenosine diphosphate (ADP), and adenosine monophosphate (AMP) concentrations were calculated from the peak area using ChromatoPack CR-4A (Shimadzu Seiki Inc., Japan). The energy charge (EC) was calculated as follows: EC = ATP + 0.5ADP/(ATP + ADP + AMP).

### Transmission Electron Microscopy

After anesthetized, 3 mouse brains were fixed with 4% paraformaldehyde perfusion, and then taken and fixed overnight at 4°C with the same fixative. The hippocampuses of the mouse brains were cut into a small block of 1 mm^3^ and put into 1% osmium tetroxide. Then the blocks were drowned into graded series of ethanol and then in acetone. Brain tissue slices were embedded with Poly/Bed 812 embedding kit (Polysciences, Warrington, PA, United States), sliced on Ultracut E ultramicrotome (Reichert, Buffalo, NY, United States), and then stained with uranyl acetate and lead citrate. The tissue sections were observed with a Hitachi H7650 transmission electron microscope (Hitachi High-Tech, Fukuoka, Japan), and images were collected with an AMT Camera System at an acceleration voltage of 80.0 kV.

### Isolation of Brain Mitochondria

Mouse cerebral mitochondria were isolated according to the method of Lai and Clark (1979). Mouse brains were homogenized in isolation buffer (225 mM mannitol, 75 mM sucrose, 1 mM EGTA, 5 mM HEPES, 2 mg/mL fat-free BSA) using a motorized Dounce homogenizer with eight up-and-down strokes. The homogenate was centrifuged at 1,500 *g* for 5 min, and the resulting supernatant was adjusted to 14% Percoll and centrifuged at 12,000 *g* for 10 min. The mitochondrial pellet was resuspended in isolation buffer and recentrifuged at 8000 *g* for 10 min. Mitochondria were finally washed and resuspended in an isolation buffer. Protein concentrations were determined by the bicinchoninic acid (BCA) protein assay kit (Pierce Chemical Co., United States) with BSA as a standard protein.

### Isolation of Phospholipids From Mitochondria of Mouse Brain

Phospholipids were isolated according to the modified methods of Bligh and Dyer (1959) and Sparagna et al. (2005). For each sample, 0.08 nmol of 1,1′,2,2′-tetramyristoyl cardiolipin (M_4_CL) was added as internal standard per 120 μg mitochondrial protein. Then methanol, chloroform, and HCl were added to mitochondrial extraction, and the mixture was vortexed for 1 min and centrifuged at 3,000 *g*. The lower organic layer was transferred to a clean glass tube and dried under a stream of nitrogen. The phospholipids extracts were resuspended in 100 μL of hexane-isopropanol (30:40, v/v).

### Tetralinoleoyl-Cardiolipin Quantitation

Tetralinoleoyl-cardiolipin (L_4_-CL) quantification was performed according to the methods of Bowron et al. (2013) with some modifications. Briefly, 10 μL of phospholipid extract was injected onto an Ultimate 3000 ultra-high-performance liquid chromatography (UPLC) (Thermo Fisher Scientific, United States). Chromatographic separation was performed on the Waters BEH C_8_ column (50 mm × 2.1 mm, 1.7 μm) at 35°C. The mobile phase A and B were 0.1% ammonium hydroxide in water and methanol, respectively, with the following gradient: 0–1.7 min, 10–90% B; 1.7–3.7 min, 90–99% B; 3.7–4.7 min, 99% B; 4.7–5.0 min, 10% B. Flow rate was 0.2 mL/min. L_4_-CL was eluted between 3.0 and 3.4 min.

A Q Exactive™ quadrupole-Orbitrap high-resolution mass spectrometer (HRMS) (Thermo Fisher Scientific, United States) was used in electrospray negative ion mode. L_4_-CL was detected by HRMS using transition with m/z 725.5 > 291.1. Collision energy for L_4_-CL was 36 eV. The L_4_-CL concentration of each sample was calculated with a calibration standard and corrected for the volume of the assay sample and its responding protein concentration.

### Acetylcholine Assay

After being anesthetized with sodium pentobarbital, the mice were killed by decapitation, and the brain was stripped on ice. After rinsing with frozen physiological saline, the brain was added with methanol and denounced in a glass homogenizer in an ice bath. The homogenate was vortexed for 30 min and centrifuged at 10000 *g* for 30 min. Discarded the supernatant, and place it in a nitrogen blower to heat and blow-dry.

The level of acetylcholine in the mouse brain was detected by using Acetylcholine Assay Kit (A105-1, Nanjing Jiancheng Bioengineering Institute, Nanjing, China) according to the manufacturer’s instructions. The entire mixture was mixed thoroughly and preserved at RT for 10 min before reading on a BioTek ELX800 Reader (BioTek, Winooski, VT, United States) at absorbance 550 nm.

### NAD^+^ Measurement

The level of nicotinamide adenine dinucleotide (NAD^+^) in the mouse brain was measured with NAD^+^/NADH Assay Kit (S0175, Beyotime Biotechnology, Shanghai, China) according to the manufacturer’s instructions. The mixture was performed to read on BioTek ELX800 Reader at absorbance 450 nm.

### Immunofluorescence and Confocal Microscopy

For immunofluorescence staining, the frozen sections were permeabilized with 0.3% Triton X-100 and blocked with 5% (vol/vol) bovine serum albumin (BSA) in PBS. Then the sections were incubated with primary antibodies, including anti-S100B antibody (1:500, AMAb91038, Atlas Antibodies AB, Stockholm, Sweden), anti-ALDH3B2 antibody (1:50, HPA045132, Atlas Antibodies AB, Stockholm, Sweden), Anti-8-Oxoguanine (8-OxoG) Antibody (1:100, MAB3560, Millipore, Sigma-Aldrich Co. LLC., CA, United States) at 4°C overnight. Secondary antibodies were either FITC- -conjugated goat anti-mouse IgG antibody (1:100, 115-095-003, Jackson ImmunoResearch Laboratories, Inc., PA, United States), Alexa Fluor^®^ 594-conjugated goat anti-rabbit IgG antibody (1:50, 111-585-003, Jackson ImmunoResearch Laboratories, Inc., PA, United States) or Alexa Fluor^®^ 594-conjugated goat anti-mouse IgG antibody (1:50, 115-585-003, Jackson ImmunoResearch Laboratories, Inc., PA, United States), and incubated for 2 h in dark at RT. After washing in PBS, the sections were mounted with DAPI-containing mounting media (C1211, APPLYGEN, Beijing, China). Immunofluorescence reactivity or DAPI was detected by Olympus IX71 microscope and Olympus FV500 confocal laser-scanning microscope (Olympus, Japan), and analyzed with Image J 1.46r.

### Measurements of Mitochondrial Membrane Potential

Isolated mitochondrial membrane potential (MMP) (Δψm) was measured with JC1-Mitochondrial Membrane Potential Assay Kit (ab113850, abcam, Cambridge, United Kingdom) according to the manufacturer’s instruction. Briefly, isolated mitochondria were mixed with a working JC-1 solution diluted with dilution buffer. Then the mixture was loaded in a read plate and detected by Synergy H1 Hybrid Multi-Mode Microplate Reader at the excitation wavelength of 535 or 475 nm and emission wavelength at 590 or 530 nm. MMP was determined by the ratio of fluorescence intensity between 590 and 530 nm.

### Mitochondrial Swelling Assay

Mitochondrial swelling was evaluated as described by Zangeneh et al. (2018). The mitochondrial swelling was determined by measuring decreased absorbance at 540 nm *via* BioTek Synergy H1 Hybrid Multi-Mode Microplate Reader. The cerebral mitochondria of mice were suspended in swelling buffer (70 mmol/L of sucrose, 230 mmol/L of mannitol, 3 mmol/L of HEPES, 2 mmol/L of Tris-phosphate, 5 mmol/L of succinate, and 1 μmol/L of rotenone). The mixture was incubated at 30°C for 1 h. The absorbance was measured at 5 and 45 min.

### Mitochondrial Respiratory Chain Complex I, II, III, and IV Enzyme Activity Assay

Mitochondrial respiratory chain complex I II, III, and IV were measured by Complex I Enzyme Activity Microplate Assay Kit (ab109721, abcam, Cambridge, United Kingdom), Complex II Enzyme Activity Microplate Assay Kit (ab109908, abcam, Cambridge, United Kingdom), MitoTox Complex II + III OxPhos Activity Assay Kit (ab109905, abcam, Cambridge, United Kingdom), and Complex IV Rodent Enzyme Activity Microplate Assay Kit (ab109911, abcam, Cambridge, United Kingdom) respectively according to the manufacturer’s recommendation. Briefly 80 μg mitochondrial proteins were loaded in a 96-well plate, and the enzyme activity was determined colorimetrically with BioTek Synergy H1 Hybrid Multi-Mode Microplate Reader (BioTek, Winooski, VT, United States) by detecting the light absorption value of the sample at 450 nm (complex I), 600 nm (complex II), and 550 nm (complex III and complex IV) respectively.

### Reactive Oxygen Species Assay

Cerebral reactive oxygen species (ROS) was assessed as described by Ali et al. (2015, 2018) previously with some modification. ROS assay was based on the oxidation of 2′7′-dichlorodihydrofluorescin diacetate (DCFH-DA) to 2′7′-dichlorofluorescein (DCF). Briefly, brain homogenate was diluted with ice-cold Lock’s buffer (pH 7.4) to a final concentration of 5 mg tissue/mL. Then the Lock’s buffer, diluted brain homogenate, and DCFH-DA (final concentration of 5 mmol/L) were mixed and incubated at RT for 15 min. The conversion of DCFH-DA to the DCF was determined by BioTek Synergy H1 Hybrid Multi-Mode Microplate Reader with excitation at 484 nm and emission at 530 nm. The ROS level was quantified from the DCF-standard curve and expressed as relative DCF pmol/mg protein.

### Lipid Peroxidation Assay

The LPO levels in the mouse brain were determined by analyzing the malondialdehyde (MDA) level. MDA was examined by using the commercial lipid peroxidation assay kit (ab118970, abcam) according to the manufacturer’s instructions. The samples were analyzed with BioTek Synergy H1 Hybrid Multi-Mode Microplate Reader with excitation at 532 nm and emission at 553 nm.

### Statistical Analysis

Statistical analysis was carried out using the SPSS program (version 20.0 for windows) (IBM, Armonk, NY, United States). Values were expressed as mean ± SEM. 18 mice were used for the MWM test, and 5 mice were used for metabolomics analysis. The escape latencies and average swimming speed in the MWM test were analyzed with two-way repeated-measures ANOVA followed by Fisher’s protected least significant difference (LSD) test for *post hoc* comparisons. Other data from behavioral assay were analyzed using one-way ANOVA followed by the LSD test for *post hoc* comparisons. The metabolomic data were transformed with Log2 for final statistical analysis. The PCA and the OPLS-DA were conducted with SIMCA-P software (Umetrics, Sweden), and another univariate analysis including independent sample *t*-test and *p*-value FDR adjust. *P* ≤ 0.05 and *P* ≤ 0.01 were considered to be statistically significant and highly significant, respectively.

## Results

### Ultra-High-Performance Liquid Chromatography Profile of Dihuang-Yinzi

**FIGURE 2:**
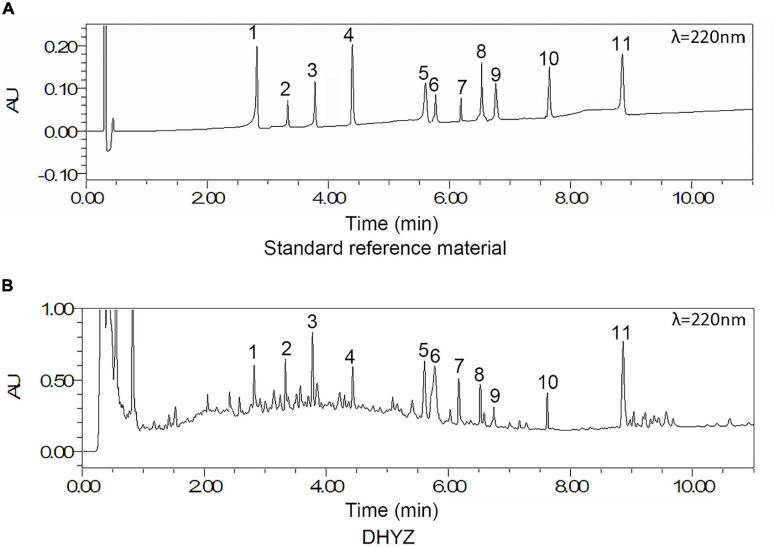
UPLC-UV chromatograms of DHYZ at 220 nm. Representative chromatograms of standard reference compound **(A)** and DHYZ **(B)** were shown respectively. Peak no.: 1. loganin, 2. echinacoside, 3. verbascoside, 4. erianin, 5. cinnamic acid, 6. tenuifolin, 7. quercetin, 8. ruscogenin, 9. kaempferol, 10. beta-sitosterol, and 11. schisandrin A.

The chromatographic fingerprint of multi-components of the DHYZ was characterized by UPLC. As shown in Figure 2, 11 ingredients in DHYZ were identified and determined by comparing them with the standard reference compounds. The names of the 11 composite ingredients and their source herbs are listed in Table 2.

**TABLE 2:** Composite ingredients and their source herbs.

Active ingredient	Chromatographic retention time (min)	Source herb
loganin	2.813	dogwood
echinacoside	3.331	cistanche, jujube
verbascoside	3.774	rehmannia, dogwood, jujube, ginger
erianin	4.433	dendrobium
cinnamic acid	5.629	cinnamon
tenuifolin	5.778	polygala
quercetin	6.272	cistanche, jujube
ruscogenin	6.515	ophiopogon
kaempferol	6.733	calamus
beta-sitosterol	7.648	morinda
schisandrin A	8.881	schisandrae

### Effects of Dihuang-Yinzi on Learning and Memory Deficits in 2× Tg-AD Mice

**FIGURE 3:**
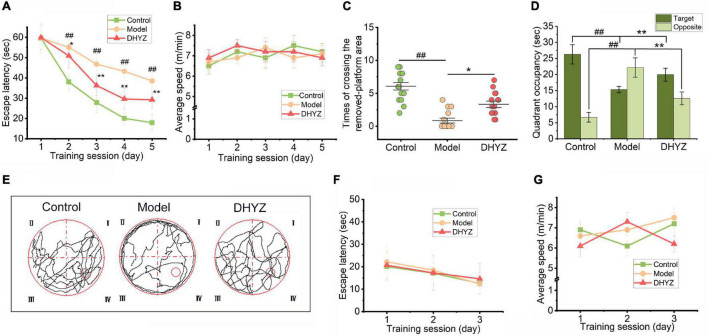
DHYZ treatment improves spatial memory of 2× Tg-AD mice in the Morris water maze (MWM) test. In the place navigation test, the escape latency **(A)** and average swimming speed **(B)** of each group were recorded. In the spatial probe test, the times of crossing the removed platform area **(C)**, the residence time of the 3 groups of mice in the target quadrant, and the opposite quadrant **(D)** were assayed, and representative images of the route of travel **(E)** were recorded. The escape latency **(F)** and average swimming speed **(G)** in the visible-platform test were measured. All data are presented as the mean ± SEM (*n* = 18 mice per group). ^##^*P* < 0.01, model *vs.* control; *P < 0.05 and **P < 0.01, DHYZ *vs.* model. AD, Alzheimer; DHYZ, Dihuang-Yinzi.

To evaluate the effects of DHYZ on spatial learning and memory ability in 2× Tg-AD mice, the MWM test was employed to assess learning capacity *via* escape latency (i.e., time spent to reach the hidden platform). As shown in Figure 3A, during the 5-day training session of the place navigation test, the average escape latency of all the three groups of mice gradually declined, and two-way ANOVA with repeated measuring revealed that there were significant differences among the three groups: day, *F*(4, 68) = 150.60, *P* < 0.0001; treatment, *F*(2, 34) = 98.79, *P* < 0.0001; day by treatment interaction, *F*(8, 136) = 9.515, *P* < 0.0001. During the training session from the 2nd to the 5th day, Non-Tg mice exhibited a shorter escape latency than 2× Tg-AD mice (day1, *P* > 0.9999; day2∼day5, *P* < 0.0001). At the same time, on the 3rd to 5th day, DHYZ treatment could significantly diminish the escape latency of 2× Tg-AD mice as compared to the mice of model group (day1, *P* = 0.4755; day2, *P* = 0.1278; day3, *P* = 0.0012; day4 and day5, *P* < 0.0001). The escape latency may also be determined by the mouse’s physical fitness. To explore whether DHYZ decreased the escape latency of mice due to the impact on their physical fitness, we measured the average swimming speed of the mice. As shown in Figure 3B, there was no significant difference in the average swimming speed among all three groups: day, *F*(4, 68) = 27.600, *P* = 0.472; treatment, *F*(2, 34) = 5.375, *P* = 0.596; day by treatment interaction, *F*(8, 136) = 3.413, *P* = 0.219. This indicates that DHYZ reduced the escape latency of 2× Tg-AD mice not by affecting their physical fitness.

In spatial probe test performed on the 6th day of the MWM test, there was a significant difference in the number of times each group of mice crossed the platform area where the platform was originally located [*F*(2,51) = 21.89, *P* < 0.0001] based on one-way ANOVA (Figure 3C). Meanwhile, DHYZ increased the number of times crossing the platform area remarkably (*P* = 0.0208). The quadrant where the platform was originally located was defined as the target quadrant (that is quadrant IV); the diagonal position of the target quadrant was defined as the opposite quadrant (that is quadrant II). As shown in Figure 3D, there were significant differences in the quadrant occupancy of the 3 groups of mice in the target quadrant [*F*(2,51) = 25.12, *P* < 0.0001] and the opposite quadrant [*F*(2,51) = 9.76, *P* < 0.0001] based on one-way *ANOVA*. Furthermore, 2× Tg-AD mice showed shorter residence time in the target quadrant (*P* < 0.0001) and longer residence time in the opposite quadrant (*P* = 0.0084) compared to Non-Tg mice. Compared with the model group, DHYZ administration dramatically increased the residence time of 2× Tg-AD mice in the target quadrant (*P* = 0.0042) and reduced the residence time in the opposite quadrant (*P* = 0.0074). The swimming trajectories of three groups of mice in the spatial probe test were shown in Figure 3E.

The escape latency of mice searching for the hidden platform in the MWM depended not only on their spatial cognition ability and physical fitness, but also on their visual ability. To determine whether there was a difference in the eyesight of each group of mice and whether this difference would affect the ability of mice to search for the platform, the visible-platform test was performed. As shown in Figures 3F,G, under the premise that the platform was visible, there was no significant difference in the escape latency among the three groups of mice: treatment, *F*(2, 34) = 0.7231, *P* = 0.4926; day by treatment interaction, *F*(4, 68) = 0.7010, *P* = 0.5939, and at the same time, there was no significant difference in their swimming speed: day, *F*(2, 34) = 0.6492, *P* = 0.5228; treatment, *F*(2, 34) = 0.9291, *P* = 0.4047; day by treatment interaction, *F*(4, 68) = 1.8190, *P* = 0.1352. These results indicated that the difference in the escape latency of the three groups of mice searching platforms in the MWM test has nothing to do with their visual acuity and physical strength.

### Network Pharmacology Analysis of Dihuang-Yinzi

**FIGURE 4:**
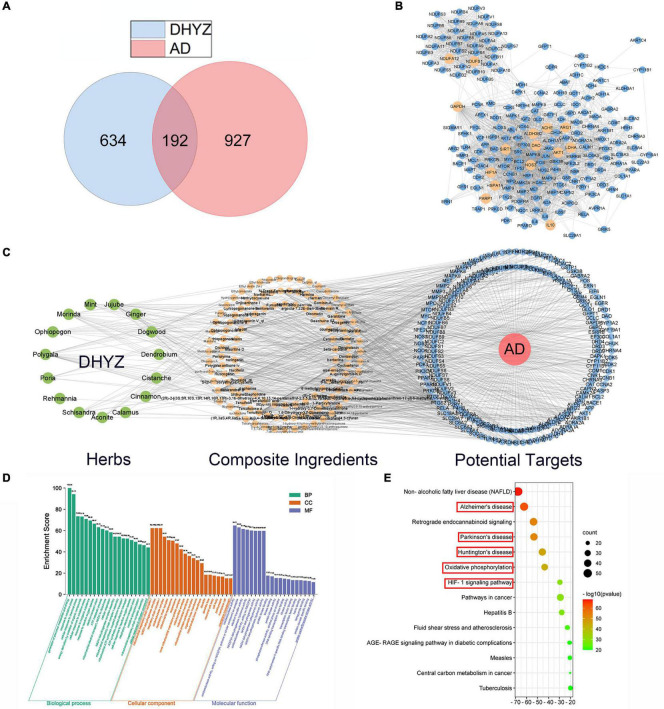
Network pharmacology analysis of DHYZ against AD. **(A)** Venn diagrams of the targets of DHYZ and AD, and common-targets of DHYZ against AD. **(B)** The protein-protein interaction network of targets of DHYZ against AD. The nodes with faint yellow color represent the hub genes. **(C)** The herbs-composite ingredients-potential targets network of DHYZ in the treatment of AD. The green, faint yellow, and blue nodes represent herbs that makeup DHYZ, composite ingredients, and the potential target genes, respectively. GO enrichment **(D)** and KEGG pathway enrichment analyses **(E)** were performed to probe the biological function and potential mechanisms of 192 potential target genes of DHYZ in treating AD. GO, gene ontology; KEGG, Kyoto Encyclopedia of Genes and Genomes; BP, biological processes; CC, cell components; MF, molecular functions; AD, Alzheimer’s disease; DHYZ, Dihuang-Yinzi.

To explore the active ingredients and potential molecular targets of DHYZ which have therapeutic effects on AD, we performed network pharmacological analysis. First, 123 composite compounds in DHYZ were retrieved from the database (Supplementary Table 1). Moreover, based on the obtained composite compounds, we identified 829 predicted targets of DHYZ. After searching the keywords of “Alzheimer” in the genecards, 1119 AD-related targets were collected. 192 common targets as potential therapeutic targets of DHYZ against AD were obtained *via* matching the predicted targets of DHYZ and AD (Figure 4A and Supplementary Table 2). All the 192 targets were normalized to their official symbols and then were input into STRING 11.0 to construct a PPI network which gave a whole view of the relationship within the targets (Figure 4B). By using the CytoHubba to calculate the hub genes, we combined the scores of 15 computational methods, then choosed the top 15 genes as the hub genes, including *NDUFA12*, *NDUFS1*, *GAPDH*, *IL10*, *AKT1*, *SIRT1*, *HIF1A*, *NOS3*, *HSPA1A*, *PARP1*, *ARG1*, *LDHA*, *ACHE*, *ALDH3B2*, and *DAO* (Supplementary Table 3). All hub genes were presented as the faint yellow nodes in the PPI network (Figure 4B), suggesting that these hub genes might be the key target genes for DHYZ to improve cognitive dysfunction in AD.

We then constructed the herbs-composite ingredients-potential targets network by Cytoscape 3.8.2 for a global view, which was constituted by 15 herbs that make up DHYZ, 123 composite ingredients, and 192 potential targets for DHYZ to treat AD (Figure 4C). We next performed GO and KEGG pathways enrichment analyses. The top-twenty enriched GO terms ranked by *p*-value in biological processes, cell components, and molecular functions are presented in Figure 4D and Supplementary Table 4. The top-five terms in GO biological processes were the generation of precursor metabolites and energy (GO:0006091), response to oxidative stress (GO:0006979), cellular response to chemical stress (GO:0062197), energy derivation by oxidation of organic compounds (GO:0015980), cellular response to oxidative stress (GO:0034599). This indicated that restoring energy metabolism and inhibiting oxidative stress are major aspects of DHYZ’s anti-AD effect. In terms of GO cellular components, the top-five enriched items were mitochondrial respiratory chain complex I (GO:0005747), NADH dehydrogenase complex (GO:0030964), respiratory chain complex I (GO:0045271), oxidoreductase complex (GO:1990204), respiratory chain complex (GO:0098803). Regarding GO molecular functions, the top-five terms were oxidoreductase activity (GO:0016491), oxidoreductase activity, acting on NAD(P)H, quinone or similar compound as acceptor (GO:0016655), electron transfer activity (GO:0009055), NAD(P)H dehydrogenase (quinone) activity (GO:0003955), oxidoreductase activity, acting on NAD(P)H (GO:0016651). This shows that DHYZ’s efficacy in treating AD involves the protection of mitochondrial function, oxidative phosphorylation, oxidoreductase system, etc. According to the KEGG enrichment analysis, the pathways affected significantly were Non-alcoholic fatty liver disease (NAFLD), AD, signaling, and other neurodegenerative diseases, such as Parkinson’s disease (PD), Huntington’s disease (HD), etc. In particular, KEGG enrichment analysis revealed that the DHYZ’s efficacy involves AD and other neurodegenerative diseases related pathways, oxidative phosphorylation, HIF-1 signaling pathway, etc. (Figure 4E and Supplementary Table 5).

### Untargeted Metabonomics Profiling of Alzheimer’s Disease Treatment by Dihuang-Yinzi

**FIGURE 5:**
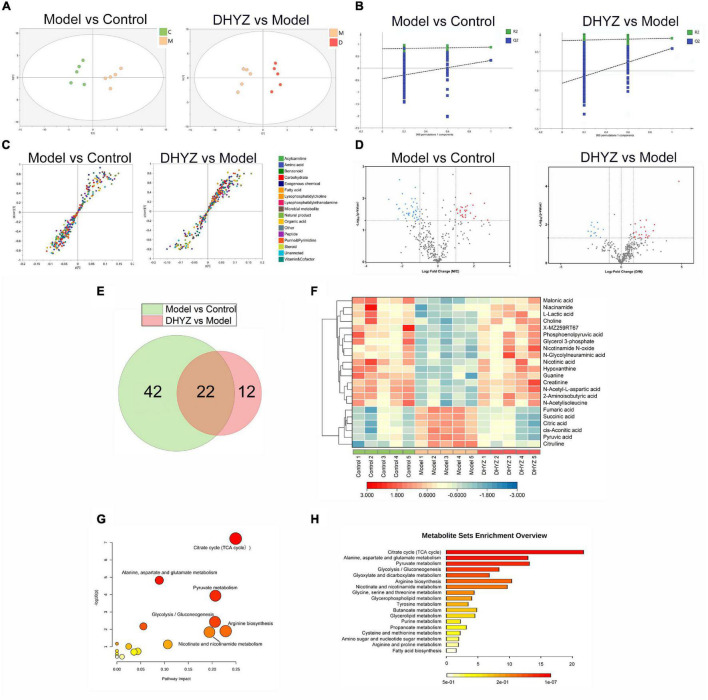
Untargeted metabonomics profiling of AD treatment by DHYZ. **(A)** Score plot of PCA modeling to maximize inter-group differentiation of metabolomics data between the model and control group and between DHYZ and model group. **(B)** 999 times permutation to test the robustness of OPLS-DA modeling between model and control group and between DHYZ and model group. **(C)** S-plot of OPLS-DA model differentiating the model *vs.* control group and the DHYZ *vs.* mode group. **(D)** Volcano plot to visualize differential metabolites of significance between the model and control group and between the DHYZ and model group. **(E)** Venn diagrams of potential metabolites between the model and control group, and between the DHYZ and model group. **(F)** The heat map of potential metabolites involved in 2× Tg-AD mice treated by DHYZ. MetaboAnalyst analysis and visualization of metabolism pathway involved in treating AD with DHYZ. **(G)** Summary of pathway analysis visualized by the dot chart. The dots represent the pathways that were matched pathway impact values from pathway topology analysis and *p*-values from pathway enrichment analysis. **(H)** KEGG enrichment analysis of metabolism pathway for associated urinary metabolites in 2× Tg-AD mice treated with DHYZ. Metabolites with *P* < 0.05 and fold change (FC) >2 were highlighted with red (up-regulated) and blue (down-regulated), respectively. C, control group; M, model group; D, DHYZ group. KEGG, Kyoto Encyclopedia of Genes and Genomes; AD, Alzheimer’s disease; DHYZ, Dihuang-Yinzi.

To identify the significantly changed metabolites of 2× Tg-AD mice with DHYZ treatment, we performed a untargeted metabolomics analysis. Principal component analysis (PCA) and OPLS-DA in multivariate analysis were performed to evaluate and determine the endogenous substances that changed significantly in rodent urine, and are also used to screen the metabolites that varied remarkably in 2× Tg-AD mice after DHYZ treatment. As shown in Figure 5A, OPLS-DA revealed that the five samples in each group are clustered together, and there was a significant distinction among samples from different groups.

The R^2^Y (cum) and Q^2^ (cum) in OPLS-DA were 0.877 and 0.542, respectively, using the data from the model *vs.* control group and 0.853 and 0.596, using the data from the DHYZ *vs.* model group, respectively. 999 times permutation tests were performed to test the robustness of OPLS-DA modeling and verify the validity of the data multivariate analysis model (Figure 5B). The permutation test showed the models were non-overfitting and reliable. These results indicated that the samples from the control group, model group, and DHYZ group could be distinguished well, and DHYZ treatment resulted in obvious metabolic variations in 2× Tg-AD mice. As shown in Figure 5C, S-plot was employed to identify altered metabolites that markedly contributed to the differences between the model and control groups and the differences between DHYZ and model groups, respectively. Moreover, volcano plots revealed that the up-regulated (red plots) and down-regulated (blue plots) metabolites were significantly different between the model and control group and between the DHYZ and model group with *P* < 0.05 and fold change (FC) >2 (Figure 5D).

Based on *P* < 0.05 and FC > 2, 64 differential metabolites were identified in the urine between the model and the control group; 44 differential metabolites were identified between the DHYZ and model groups. Finally, 22 common metabolites were identified as differential metabolites that DHYZ affected 2× Tg-AD mice in urine (Figure 5E and Table 3). To further visualize the variation in metabolites among the control, model, and DHYZ group, we plotted the heat map to show the 22 differential metabolites changed among the 3 groups (Figure 5F). Notably, compared with the control group, most of the differential metabolites that changed in the model group were reversed after DHYZ intervention, indicating that DHYZ could ameliorate metabolic perturbation in 2× Tg-AD mice.

**TABLE 3:** Differential metabolites in the urine of 2× Tg-AD mice treated by DHYZ.

No	Class	Metabolite	HMDB	m/z	Model *vs.* Control		DHYZ *vs.* Model	
					*P*	Log_2_FC	*P*	Log_2_FC
1	Amino acid	Citrulline	HMDB0000904	176.10	0.0018	1.5410	0.0029	−1.0654
2	Amino acid	N-Acetylisoleucine	HMDB0061684	174.11	0.0187	−1.1439	0.0294	1.1873
3	Amino acid	N-Acetyl-L-aspartic acid	HMDB0000812	174.04	0.0326	−1.2431	0.0244	1.2255
4	Amino acid	Creatinine	HMDB0000562	114.06	0.0311	−1.2653	0.0456	1.7897
5	Amino acid	Choline	HMDB0000097	104.10	0.0311	−1.2834	0.0257	1.0508
6	Carbohydrate	N-Glycolylneuraminic acid	HMDB0000833	324.09	0.0231	−1.5321	0.0312	2.0584
7	Carbohydrate	L-Lactic acid	HMDB0000190	89.02	0.0166	−1.5633	0.0376	1.5230
8	Carbohydrate	Phosphatidate	HMDB00636	171.00	0.0217	−1.6789	0.0313	1.4140
9	Organic acid	Pyruvic acid	HMDB0000243	87.01	0.0234	1.6462	0.0101	−1.6273
10	Organic acid	cis-Aconitic acid	HMDB0000072	173.01	0.0264	1.8495	0.0183	−1.6000
11	Organic acid	Phosphoenolpyruvic acid	HMDB0000263	166.96-166.98	0.0364	−2.1785	0.0313	1.8467
12	Organic acid	Citric acid	HMDB0000094	191.02	0.0353	2.2725	0.0257	−2.1395
13	Organic acid	Fumaric acid	HMDB0000134	115.00	0.0376	2.6311	0.0470	−1.7610
14	Organic acid	2-Aminoisobutyric acid	HMDB0001906	102.05	0.0218	−1.1071	0.0260	1.0385
15	Organic acid	Succinic acid	HMDB0000254	117.02	0.0187	2.5398	0.0333	−2.3221
16	Organic acid	Methylmalonate	HMDB00202	103.00	0.0045	−1.2553	0.0100	1.1760
17	Purine and Pyrimidine	Guanine	HMDB0000132	152.06	0.0072	−1.9736	0.0061	1.5794
18	Purine and Pyrimidine	Hypoxanthine	HMDB0000157	137.05	0.0132	−1.3563	0.0425	1.1240
19	Unannotated	X-MZ259RT67		259.10	0.0103	−1.2613	0.0488	1.0559
20	Vitamin and Cofactor	Nicotinamide N-oxide	HMDB0002730	139.05	0.0305	−1.3816	0.0155	1.8438
21	Vitamin and Cofactor	Nicotinic acid	HMDB0001488	124.04	0.0280	−1.1512	0.0396	1.0409
22	Vitamin and Cofactor	Niacinamide	HMDB0001406	123.06	0.0335	−1.2190	0.0382	1.0404

To further reveal the key metabolic pathways involved in process of the AD treatment by DHYZ, we performed KEGG pathway enrichment by using the MetaboAnalyst 5.0. Based on pathway impact >0.1, 6 signaling pathways were enriched: TCA cycle, pyruvate metabolism, glycolysis/gluconeogenesis, arginine biosynthesis, nicotinate, nicotinamide metabolism, and glycerophospholipid metabolism (Figures 5G,H). The key differential metabolites involved in these pathways were pyruvate, succinate, fumarate, citrate, phosphoenolpyruvate, cis-aconitate, (s)-lactate, L-citrulline, nicotinate, nicotinamide, choline, and sn-glycerol-3-phosphate. All pathways with *P* < 0.05 or pathway impact >0.1 were listed in Table 4.

**TABLE 4:** Pathways were affected significantly after DHYZ intervention with *P* < 0.05 or pathway impact >0.1.

Pathway Name	Match Status	*p*	−log(p)	Holm p	FDR	Impact	Details
tricarboxylic acid cycle (TCA cycle)	6/20	5.9036E-8	7.2289	4.959E-6	4.959E-6	0.24929	KEGG
Alanine, aspartate and glutamate metabolism	5/28	1.4882E-5	4.8273	0.0012352	6.2506E-4	0.08894	KEGG SMP
Pyruvate metabolism	4/22	1.1493E-4	3.9396	0.009424	0.0032179	0.20684	KEGG SMP
Glycolysis/Gluconeogenesis	3/26	0.0036879	2.4332	0.29872	0.077447	0.20594	KEGG SMP
Glyoxylate and dicarboxylate metabolism	3/32	0.0067053	2.1736	0.53643	0.11265	0.05556	KEGG
Arginine biosynthesis	2/14	0.012543	1.9016	0.99086	0.17237	0.22843	KEGG
Nicotinate and nicotinamide metabolism	2/15	0.014364	1.8427	1.0	0.17237	0.1943	KEGG SMP
Glycerophospholipid metabolism	2/36	0.073657	1.1328	1.0	0.68747	0.10675	KEGG

### Integrated Analysis of Metabolomics and Network Pharmacology in the Treatment of Alzheimer’s Disease by Dihuang-Yinzi

**FIGURE 6:**
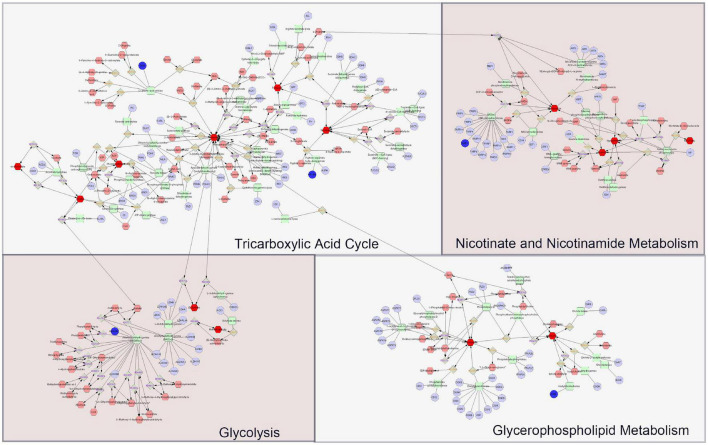
The compound-reaction-enzyme-gene networks based on the key metabolites and targets of DHYZ against AD. The faint red hexagons, gray diamonds, green round rectangle, and purple circles represent the compounds, reactions, proteins, and genes, respectively. The scarlet hexagons and dark blue circles represent the key metabolites and target genes of DHYZ against AD. AD, Alzheimer’s disease; DHYZ, Dihuang-Yinzi.

As it is aimed to obtain a systematic and comprehensive view of mechanisms of DHYZ against AD, we constructed an interaction network based on metabolomics and network pharmacology (Figure 6). Differential metabolites were imported into the MetScape plugin in Cytoscape to construct the compound-reaction-enzyme-gene networks. By matching the hub genes screened in network pharmacology analysis with the differential metabolites in MetScape analysis, we identified 5 key target genes, including *DAO*, *HIF1A*, *ALDH3B2*, *PARP1*, and *ACHE*. 14 related key metabolites were identified as follows: cis-aconitic acid, citric acid, phosphoenolpyruvic acid, pyruvic acid, fumaric acid, succinic acid, L-lactic acid, malonic acid, nicotinamide, guanine, hypoxanthine, nicotinic acid, glycerol-3-phosphate, and choline. The 4 key pathways were the TCA cycle, glycolysis, nicotinate and nicotinamide metabolism, and glycerophospholipid metabolism (Table 5). These data revealed that all the 5 key target genes, 14 metabolites, and 4 key metabolic pathways may play essential roles in the therapeutic effect of DHYZ against AD.

**TABLE 5:** The information of key targets, metabolites, and metabolic pathways.

Related pathway	Target gene	Key metabolite
TCA cycle	*DAO*, *HIF1A*	cis-Aconitic acid, Citric acid, Phosphoenolpyruvic acid, Pyruvic acid, Fumaric acid, Succinic acid
Glycolysis	*ALDH3B2*	L-Lactic acid, Methylmalonic acid
Nicotinate and Nicotinamide Metabolism	*PARP1*	Nicotinamide, Guanine, Hypoxanthine, Nicotinic acid
Glycerophospholipid Metabolism	*ACHE*	Phosphatidate, Choline

### Effects of Dihuang-Yinzi on Glycerophospholipid Metabolism and the Structure and Function of Mitochondria

**FIGURE 7:**
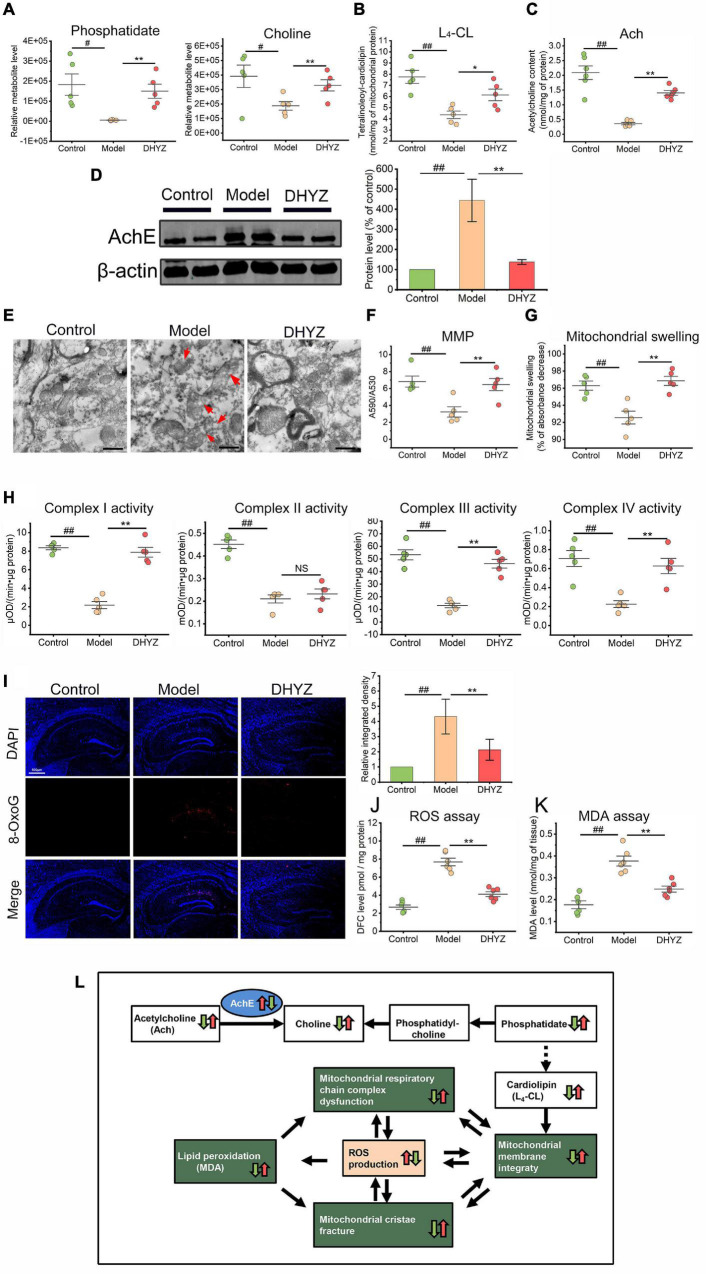
The preservation effects of DHYZ on the glycerophospholipid metabolism and the structure and function of mitochondria. **(A)** Metabolomics analysis illustrating the relative levels of urine phosphatidate and choline. The level of L_4_-CL (*n* = 5) **(B)** and Ach (*n* = 6) **(C)** in the brain of 2× Tg-AD mice were determined with LC-MS and fluorescence chemistry assay, respectively. **(D)** The AchE expression was detected by Western blotting (*n* = 3). **(E)** Representative electron micrographs of hippocampal tissues of mice of the control, model, and DHYZ group. Red arrows denote the lesions of mitochondrial ultramicrostructure (magnification ×40 000, scale bar = 500 nm). MMP **(F)** and mitochondrial swelling **(G)** were measured to assess the integrity of the mitochondrial membrane. **(H)** The activities of respiratory chain complexes I, II, III, and IV were assessed to evaluate the mitochondrial function. **(I)** Representative images of immunofluorescence staining of 8-OxoG (red, Alexa Fluor^®^ 594; blue, DAPI) and the corresponding histogram of 8-OxoG relative intensity in hippocampus regions of mice of the control, model, and DHYZ group (*n* = 5 mice per group; magnification ×40, scale bar = 500 μm). **(J)** The scatter dot plot diagram of ROS level is expressed as DFC level. **(K)** The LPO was determined by measuring the MDA level. **(L)** Diagram depicting alteration in glycerophospholipid metabolism and the structure and function of mitochondria in AD mouse in response to DHYZ treatment. White squares represent metabolites, green squares represent mitochondrion function, blue ellipses represent target proteins or key enzymes. Red up-arrows and green down-arrows indicate up-regulation and down-regulation, respectively. Left arrows represent model *vs.* control; right arrows represent DHYZ *vs.* model. All data are presented as the mean ± SEM. ^#^*P* < 0.05 and ^##^*P* < 0.01, model *vs.* control; *P < 0.05 and **P < 0.01, DHYZ *vs.* model. AD, Alzheimer’s disease; DHYZ, Dihuang-Yinzi; L_4_-CL, tetralinoleoyl-cardiolipin; Ach, acetylcholine; AchE, acetylcholinesterase; MMP, mitochondrial membrane potential; 8-OxoG, 8-Oxoguanine; ROS, reactive oxygen species; LPO, lipid peroxidation; MDA, malondialdehyde.

As confirmed by metabolomics analysis, DHYZ could elevate the relative abundance of phosphatidate (*P* < 0.01) and choline (*P* < 0.01) in the urine of 2× Tg-AD mice (Figure 7A). Since phosphatidate is an important precursor for the synthesis of cardiolipin (CL) which is the signature phospholipid of mitochondrial membranes, we determined the content of Tetralinoleoyl-cardiolipin (L_4_-CL), the major CL species, in the mouse brains. The cerebral L_4_-CL level in 2× Tg-AD mice decreased significantly (*P* < 0.01), and DHYZ could reverse this trend remarkedly (*P* < 0.01) (Figure 7B). Acetylcholine (Ach), a precursor of choline, is the cerebral neurotransmitter most closely related to learning and memory. As shown in Figure 7C, the Ach level in the brain of mice in model group decreased remarkably compared to the control group (*P* < 0.01), and DHYZ treatment increased the cerebral Ach level in 2× Tg-AD mice dramatically (*P* < 0.01). Acetylcholinesterase (AchE) is a clinically proven therapeutic target for AD treatment. Using Western blotting assay, we confirmed that DHYZ could down-regulate AchE expression (*P* < 0.01) in 2× Tg-AD mice (Figure 7D). These results were consistent with the effect of DHYZ on improving the learning and memory of AD mice as confirmed in behavioral tests.

As the signature phospholipid of mitochondrial membranes, CL is closely related to the function and structure integrity of mitochondria, and a decrease in the CL level would results in serious destruction to the mitochondrial membrane disruption (Kameoka et al., 2018). As shown in Figure 7E, we observed the obvious swelling, membrane structure destruction, cristae breakage in mitochondria in 2× Tg-AD mice. On the contrary, DHYZ could protect the mitochondria from those ultrastructure destructions. In line with this, DHYZ increased MMP and depressed mitochondrial swelling in 2× Tg-AD mice (Figures 7F,G).

The OxPhos system consists of 5 enzymatic complexes (Complexes I to V) which form the mitochondrial respiratory chain are embedded in the inner mitochondrial membrane. The OxPhos is strongly associated with mitochondrial membrane integrity. To study the impact of DHYZ on OxPhos, we measured the activities of Complex I ∼ IV. Consistent with the protecting role of DHYZ in mitochondrial membrane integrity, DHYZ treatment markedly improved the activities of the Complex I, III, and IV respectively (*P* < 0.01), but had no significant effect on the activity of the Complex II (Figure 7H).

Mitochondria are the major source and producer of intracellular ROS. Overproduction of ROS and the resultant oxidative stress are the indicators of mitochondrial dysfunction. To investigate whether DHYZ could inhibit oxidative stress, we assessed oxidative damage by quantifying 8-Oxoguanine (8-OxoG) adducts and the conversion of DCFH-DA to the DCF in the brain of AD mice. As shown in Figures 7I,J, 2× Tg-AD mice exhibited excessive ROS production, especially in the hippocampus, as compared with the control mice (*P* < 0.01), and DHYZ dramatically eliminate ROS overload in the brain of AD mice (*P* < 0.01). Excessive production of ROS results in lipid peroxidation (LPO) and malondialdehyde (MDA) generation, the latter is the hallmark of oxidative damage on the cellular membrane system, including the mitochondrial membrane. As expected, we observed that the MDA level was significantly elevated compared to the control group (*P* < 0.01) due to oxidative injury, and DHYZ treatment remarkably decreased the MDA level (*P* < 0.01) (Figure 7K).

Taken together, DHYZ improved glycerophospholipid metabolism and increased CL levels, thereby protecting mitochondrial membrane integrity and mitochondrial function, and reducing ROS overproduction and oxidative damage. At the same time, DHYZ restored Ach levels and improved cognitive performance by inhibiting AchE activity (Figure 7L).

### Dihuang-Yinzi Adjusted Nicotinate and Nicotinamide Metabolism and Promoted Glycolysis

**FIGURE 8:**
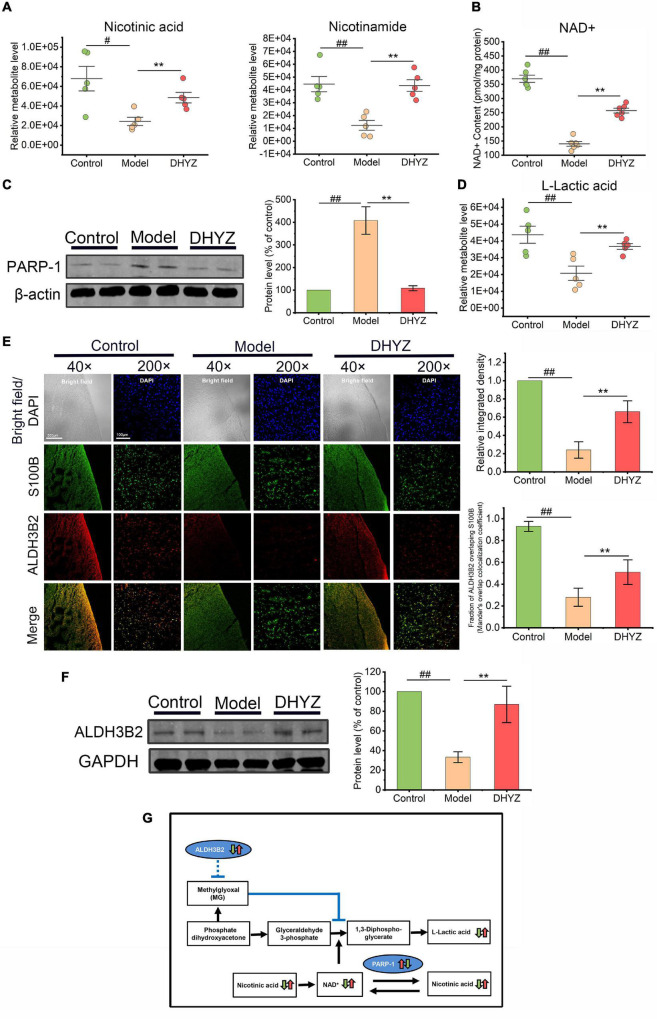
The preservation effects of DHYZ on nicotinate and nicotinamide Metabolism and glycolysis. **(A)** Metabolomics analysis illustrating the relative levels of urine nicotinic acid and nicotinamide (*n* = 5). **(B)** The scatter dot plot diagram of the level of NAD^+^ in the brain of mice in three groups was determined by photochemical assay (*n* = 6). **(C)** The protein level of PARP-1 was measured by western blot analysis (*n* = 3). **(D)** The relative level of urine L-lactic acid as illustrated in metabolomics analysis (*n* = 5). **(E)** Representative images of immunofluorescence staining of ALDH3B2 (Red, Alexa Fluor^®^ 594) and S100B (Green, FITC) (*n* = 5 mice per group; magnification ×40, scale bar = 500 μm; ×200, scale bar = 100 μm); the corresponding histogram of ALDH3B2 relative intensity in mouse cerebral cortex, and Mander’s overlap colocalization coefficient of ALDH3B2 and S100B (*n* = 5). **(F)** The protein level of ALDH3B2 was measured by western blot analysis (*n* = 3). **(G)** Diagram depicting preservation effects of DHYZ on nicotinate/nicotinamide Metabolism and glycolysis in 2× Tg-AD mice. All data are presented as the mean ± SEM. ^#^*P* < 0.05 and ^##^*P* < 0.01, model *vs.* control; *P < 0.05 and **P < 0.01, DHYZ *vs.* model. AD, Alzheimer’s disease; DHYZ, Dihuang-Yinzi; NAD+, nicotinamide adenine dinucleotide; PARP-1, Poly [ADP-ribose] polymerase 1; ALDH3B2, aldehyde dehydrogenase 3 family member B2.

As previously demonstrated by metabolomic analysis, the nicotinate and nicotinamide metabolism is one of the key metabolism pathways for DHYZ against AD. As shown in Figure 8A, the relative abundance of nicotinic acid (*P* < 0.05) and nicotinamide (*P* < 0.01) decreased in the urine of 2× Tg-AD mice. In line with this, there was a significant decrease in the level of nicotinamide adenine dinucleotide (NAD^+^) in the brain of 2× Tg-AD mice (*P* < 0.01) (Figure 8B). Postintervention, DHYZ markedly increased the levels of urinal nicotinic acid (*P* < 0.01) and nicotinamide (*P* < 0.01), and cerebral NAD^+^ content (*P* < 0.01) in 2× Tg-AD mice. Besides, the depletion of intracellular NAD^+^ might also result from the upregulation of Poly [ADP-ribose] polymerase 1 (PARP-1), we determined PARP-1 expression in the brain of 2× Tg-AD mice by Western blotting. As shown in Figure 8C, PARP-1 level elevated dramatically in 2× Tg-AD mice as compared to the control mice (*P* < 0.01), and DHYZ markedly depressed the level of PARP-1 in 2× Tg-AD mice (*P* < 0.01).

In glycolysis, NAD^+^ is served as an essential coenzyme for glyceraldehyde-3-phosphate dehydrogenase which catalyzes the conversion of glyceraldehyde-3-phosphate to 1,3-diphosphoglycerate (Ju et al., 2017). The levels of L-lactic acid, an important end product of glycolysis, in the urine of 2× Tg-AD mice were significantly reduced as shown by metabolomic analysis (*P* < 0.01), which might be partly due to NAD^+^ depletion. DHYZ remarkably elevated the level of L-lactic acid (*P* < 0.01), and thus promote glycolysis in 2× Tg-AD mice (Figure 8D). As one of the critical targets of DHYZ against AD, ALDH3B2 expression decreased significantly in the brain of 2× Tg-AD mice as compared to the control mice (*P* < 0.01), which was detected with fluorescence immunoassay and Western blotting (Figures 8E,F). In contrast, DHYZ could upregulate the cerebral ALDH3B2 expression in 2× Tg-AD mice (*P* < 0.01), and this was consistent with the predominantly glycolysis-promoting role of DHYZ (Figures 8E,F). Furthermore, as shown in Figure 8E, ALDH3B2 colocalized to S100B, a marker of astrocytes, in control mice. This implies that ALDH3B2 was mainly expressed in astrocytes that are the main sites of glycolysis.

Taken together, we conclude that there was an obvious disturbance in nicotinate/nicotinamide metabolism and glycolysis in 2× Tg-AD mice. DHYZ could ameliorate this chaos by elevating the abundance of nicotinic acid and nicotinamide and restoring the level of NAD^+^ by depressing the PAPR-1. Furthermore, DHYZ promoted glycolysis by upregulating ALDH3B2, improving glyceraldehyde-3-phosphate conversion, and elevating the level of L-lactic acid which is an important energy substance produced by glycolysis and delivered by astrocytes to neurons *via* astrocyte-neuron lactate shuttle (ANLS) (Figure 8G).

### Dihuang-Yinzi Improved Tricarboxylic Acid Cycle and Promoted Energy Production

**FIGURE 9:**
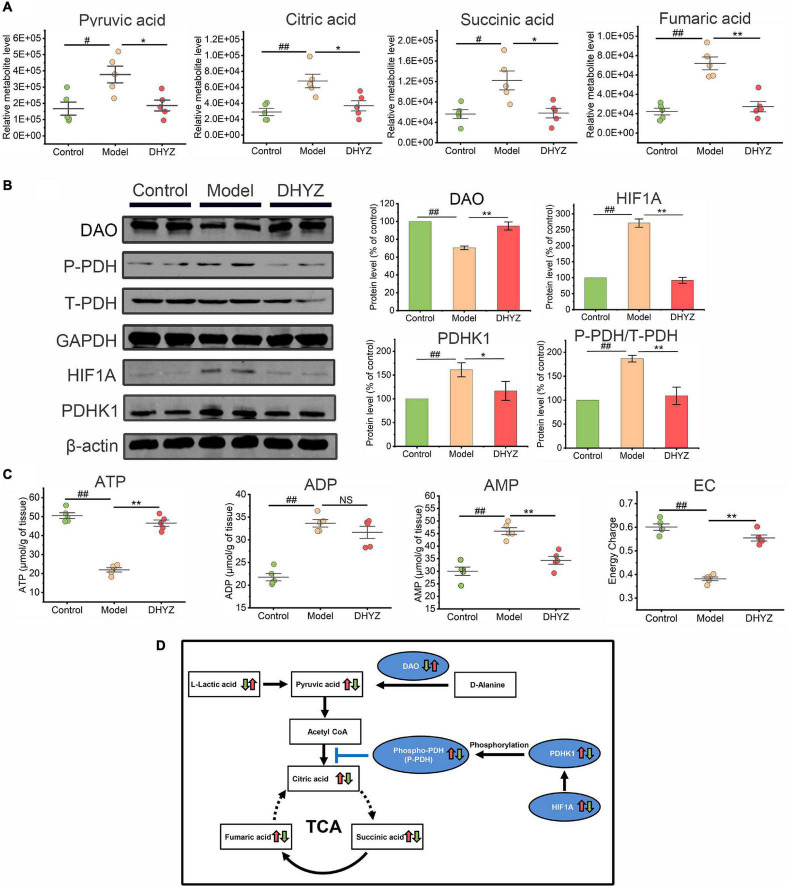
The preservation effects of DHYZ on the tricarboxylic acid (TCA) cycle. **(A)** Metabolomics analysis illustrating the relative levels of urine pyruvic acid, citric acid, succinic acid, and fumaric acid (*n* = 5). **(B)** The protein levels of DAO, phosphorylation of PDH, total PDH, HIF1A, and PDHK1 were measured by western blot analysis (*n* = 3). **(C)** The levels of ATP, ADP, and AMP in the brain of mice in 3 groups was determined by reverse-phase HPLC assay (*n* = 5). The energy charge (EC) was calculated as EC = ATP + 0.5ADP/(ATP + ADP + AMP). **(D)** Diagram depicting of protective effects of DHYZ on the TCA cycle in 2× Tg-AD mice. All data are presented as the mean ± SEM. ^#^*P* < 0.05 and ^##^*P* < 0.01, model *vs.* control; *P < 0.05 and **P < 0.01, DHYZ *vs.* model. AD, Alzheimer’s disease; DHYZ, Dihuang-Yinzi; DAO, d-amino-acid oxidase; HIF1A, hypoxia-inducible factor 1-alpha; PDHK1, pyruvate dehydrogenase kinase 1; T-, P-PDH, total, or phosphorylated pyruvate dehydrogenase; ATP, adenosine triphosphate; ADP, adenosine diphosphate; AMP, adenosine monophosphate; EC, energy charge.

Given that DHYZ ameliorated the structural and functional impairments of the mitochondria, we next investigated whether DHYZ could also modulate and preserve the TCA cycle, oxidative phosphorylation pathways, and related energy generation process that occurs in the mitochondria. Metabolomics analysis demonstrated that the contents of 4 intermediate metabolites in the TCA cycle in the urine of 2× Tg-AD mice increased remarkably, including pyruvic acid (*P* < 0.05), citric acid (*P* < 0.01), succinic acid (*P* < 0.05), and fumaric acid (*P* < 0.01) (Figure 9A). The obvious accumulation of the 4 intermediate metabolites indicated that the pronounced disturbance occurred in TCA cycle of 2× Tg-AD mice. On the contrary, DHYZ could decrease the level of urine pyruvic acid (*P* < 0.05), citric acid (*P* < 0.05), succinic acid (*P* < 0.05), and fumaric acid (*P* < 0.01) in 2× Tg-AD mice dramatically (Figure 9A).

D-amino-acid oxidase (DAO) is one of the potential targets of DHYZ against AD as the bioinformatics analysis revealed. We further confirmed the expression of DAO in the brain of 2× Tg-AD mice by using Western blotting. As shown in Figure 9B, the DAO level in the brain of 2× Tg-AD mice dramatically decreased compared to the control mice (*P* < 0.01). In contrast, DHYZ significantly upregulated DAO expression in 2× Tg-AD mice (*P* < 0.01). The upregulation of hypoxia-inducible factor 1-alpha (HIF1A) results in increased expression of pyruvate dehydrogenase kinase 1 (PDHK1), which leads to phosphorylation of pyruvate dehydrogenase (PDH) and depresses the entry of pyruvate into the TCA cycle (Lai et al., 2013). DHYZ markedly reduced the levels of HIF1A (*P* < 0.01) and PDHK1 (*P* < 0.05), and depressed the phosphorylation of PDH (*P* < 0.01), consistent with the decrease of pyruvic acid level which means more pyruvic acid was utilized and entered the TCA cycle in 2× Tg-AD mice.

The TCA cycle is the core pathway of OxPhos which is the main way to meet the high energy demand of the central nervous system (CNS). To determine whether the beneficial effects of DHYZ on the TCA cycle could result in improved energy supplement in the brain of 2× Tg-AD mice, we measured the level of the cerebral adenosine triphosphate (ATP), adenosine diphosphate (ADP), and adenosine monophosphate (AMP). As can be seen in Figure 9C, DHYZ treatment significantly increased the levels of ATP (*P* < 0.01) and EC (*P* < 0.01). Consistent with this result, the AMP level decreased remarkably in DHYZ-treated 2× Tg-AD mice (*P* < 0.01). Interestingly, despite the marked increase in the ADP level (*P* < 0.01) in 2× Tg-AD mice compared to the control mice, there was no significant change in ADP level (*P* > 0.05) in 2× Tg-AD mice upon DHYZ treatment.

Collectively, DHYZ increased the L-lactic acid level to enhance the supplement of the energy substrate of the TCA cycle. DHYZ down-regulates HIF1A and PDHK1, thereby inhibiting the phosphorylation of PDH and promoting the entry of pyruvate into the TCA cycle. Besides, DHYZ elevated the expression of DAO which converted D-alanine to pyruvic acid, and promoted the potential supplementary pyruvic acid into the TCA cycle. At the same time, the levels of pyruvic acid, citric acid, succinic acid, and fumaric acid decreased, and the level of ATP increased dramatically in 2× Tg-AD mice upon DHYZ treatment, indicating its facilitating role in the TCA cycle and related energy metabolism (Figure 9D).

## Discussion

**FIGURE 10:**
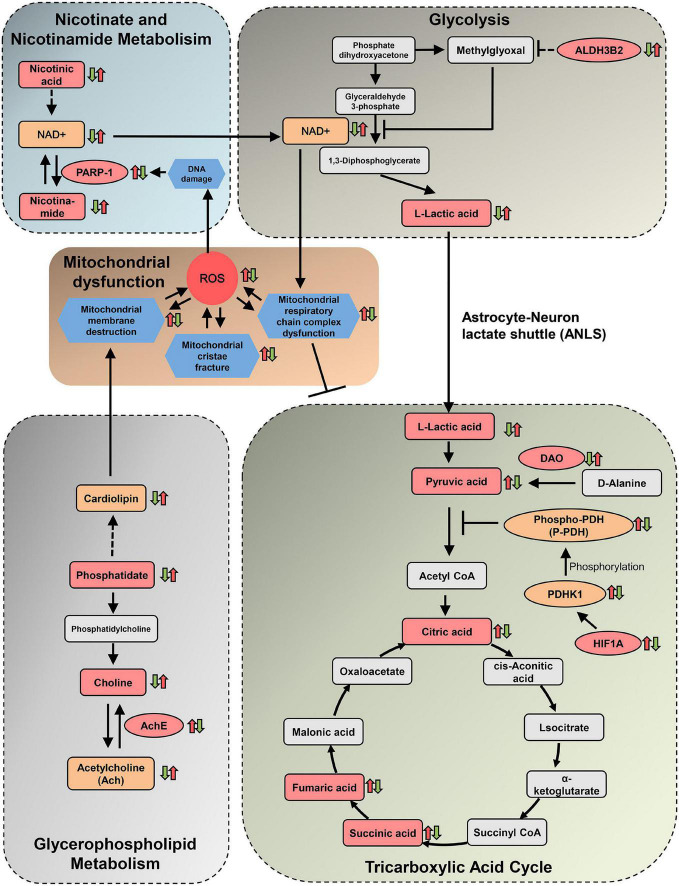
Diagram depicting protective effects of DHYZ on oxidative stress and related metabolism. The squares represent metabolites, the dark pink squares represent urine differential metabolites revealed by metabolomics analysis, the orange squares represent cerebral metabolites confirmed by other experimental verification. The dark pink ellipses represent target proteins of DHYZ against AD, the orange ellipses represent key enzymes regulated by DHYZ. Red up-arrows and green down-arrows indicate up-regulation and down-regulation, respectively. Left arrows represent model *vs.* control; right arrows represent DHYZ *vs.* model. NAD^+^, nicotinamide adenine dinucleotide; PARP-1, Poly [ADP-ribose] polymerase 1; ALDH3B2, aldehyde dehydrogenase 3 family member B2. AchE, acetylcholinesterase; DAO, d-amino-acid oxidase; HIF1A, hypoxia-inducible factor 1-alpha; PDHK1, pyruvate dehydrogenase kinase 1; T-, P-PDH, total, or phosphorylated pyruvate dehydrogenase; ROS, reactive oxygen species.

As the most common form of severe dementia, AD has complex and confusing pathogenesis. In the present study, we integrated the metabolomics and network pharmacology analysis to investigate the comprehensive relationship of the herbal ingredients, their crucial targets, differential metabolites, and related metabolic pathways. Our data presented here exhibited a systematical mechanism of DHYZ against AD, by which DHYZ could improve the cognition in 2× Tg-AD mice by ameliorating the disturbance of the 4 key energy-related metabolism pathways. The 4 key energy-related metabolic pathways targeted by DHYZ were linked by mitochondrial damage and the resultant ROS overload, forming an interconnected and complete network system (Figure 10). The results of the present study showed that DHYZ exhibited therapeutic efficacy against AD *via* the synergistic effect of multiple targets and multiple pathways. First, DHYZ improved the glycerophospholipid metabolism and depressed the resultant mitochondrial membrane disruption which contributes to OxPhos decline and the excess production of ROS. ROS overproduction might in turn cause further mitochondrial damage and DHYZ blocked the vicious circle of ROS overload and mitochondrial dysfunction. Secondly, overproduced ROS led to DNA damage which subsequently upregulated PARP-1 expression. DHYZ depressed PARP-1 and the resulting NAD^+^ depletion which contributed to the disturbance of nicotinate/nicotinamide metabolism and glycolysis. The third, DHYZ promoted the glycolysis and inhibited ROS-induced mitochondrial dysfunction, in turn, improved TCA cycle and energy supplement.

Dihuang-Yinzi is a well-accepted traditional Chinese herbal prescription which is widely used for the treatment of various aging-related diseases and neurological deficits, such as AD, Parkinson’s disease, amyotrophic lateral sclerosis, arteriosclerosis, aging-related stroke, etc. (Hu et al., 2009; Qiu et al., 2016; Lee et al., 2021). The effectiveness of DHYZ has been proven *via* clinical verification for a long time (Lee et al., 2021). In the present study, we investigated and evaluated the effects of DHYZ on spatial memory and cognition of 2× Tg-AD mice by MWM. DHYZ ameliorated cognitive impairment and memory dysfunction in 2× Tg-AD mice. Taking into account the interference of physical and visual capacity of 2× Tg-AD mice on cognitive function assessment, we confirmed that DHYZ has no significant effect on the physical fitness and visual acuity of 2× Tg-AD mice through visible-platform tests and measuring the swimming speed of each mouse.

Alzheimer’s disease is a progressive, irreversible, complex, and multifactorial neurodegenerative disease that lacks a curative treatment. The complexity of AD is a key impediment in the search for effective drug targets to treat or reverse AD-associated cognitive decline. As a consequence, the exploration of the biomarker for early diagnosis and the overall assessment of drug efficacy would be extremely advantageous to controlling AD progression and improving therapeutic outcomes. Metabolomics provides us with a powerful tool to detect perturbation in metabolites that reflect the variation of the downstream biological process of genomics, transcriptomics, and proteomics, and thus it can more accurately reflect the distinction of biological profiles between the “diseased” state as compared with the “healthy” one. For these reasons, several studies focusing on metabolome in AD have been performed over the last 5 years (Toledo et al., 2017; Varma et al., 2018; Arnold et al., 2020; Huo et al., 2020). For clinical purposes, the urinary metabolomic analysis has many advantages compared with other metabolomics analysis, such as non-invasiveness, sensitivity, convenience, and speed, etc., so it has unique predominant in AD diagnosis, drug treatment evaluation, and pathogenesis research, etc. (Khamis et al., 2017; Tynkkynen et al., 2019). Given that the pattern of single target drug treatment almost hardly to meet the requirement of the treatment for complex disease, traditional Chinese herbal prescription has become a new trend in the treatment of AD for its advantages of multi-component and multi-target with the little side effect (Yang et al., 2017; Pei et al., 2020). Metabolomics coupled with network pharmacology provides us with a systematic and novel insight of revealing the treatment of complex diseases like AD by herbal formula and provides new prospects of the potential purposeful drugs to treat AD. In the present study, we performed the urinary metabolomics coupled with network pharmacology analysis to investigate the systematical mechanism of DHYZ acting on AD through multimolecule, multitarget, and multipathway. Our results present here represented a research paradigm of AD treatment by multicomponent drugs, such as traditional Chinese herbal prescription.

As an important lipid component of mammalian cell membranes, glycerophospholipid is the most abundant type of phospholipids *in vivo* (van Meer and de Kroon, 2011). In view of this, glycerophospholipid metabolism plays a crucial role in maintaining the normal ultrastructure and function of organelles, including mitochondria. Besides, glycerophospholipids also play an important role in material transport, signal transduction, and protein function (van Meer and de Kroon, 2011; Bohdanowicz and Grinstein, 2013). CL is a specific phospholipid that composes the inner mitochondrial membrane, which is required for maintaining mitochondrial function and the optimal activity of many mitochondrial enzymes in cellular energy metabolism (Pointer and Klegeris, 2017; Chao et al., 2019). In the CNS, CL exists in neurons and glial cells, and participates in regulating its metabolic process, supporting mitochondrial function and promoting the vitality of brain cells (Pointer and Klegeris, 2017). Studies have found that the CL level significantly decreased in the brains of elderly people and AD patients (Petrosillo et al., 2008; Pointer and Klegeris, 2017). The content of mitochondrial CL in brain of senescent rats was significantly reduced by 26% compared to young rats (Ruggiero et al., 1992). In line with these results, our metabolomics analysis confirmed that the levels of CL and phosphatidate, the key substance for the CL synthesis, decreased dramatically in 2× Tg-AD mice. Furthermore, our data presented here revealed the significant reduction in the content of the cerebral L_4_-CL, a major CL species in the brain. Notably, DHYZ administration promoted glycerophospholipid metabolism by increasing the levels of phosphatidate, CL, and L_4_-CL in 2× Tg-AD mice. In previous studies, we found DHYZ could improve cognition in transgenetic AD mice and AD rats with the intracerebroventricular injection of amyloid-β peptide by ameliorating mitochondrion disturbance (Ma et al., 2012; Huang et al., 2018; Yan et al., 2018). Consistent with these findings, DHYZ could preserve the structure and function of the mitochondria *via* ameliorating the mitochondrial membrane destruction and maintaining mitochondrial integrity.

A large fraction of the total intracellular ROS is generated during the process of OxPhos in the inner mitochondrial membrane (Fontana et al., 2020). The destruction and dysfunction of mitochondria weaken enzymatic activities of the respiratory chain complex I, II, III, and IV, which are crucial for the OxPhos, and result in the abundant release of ROS in the AD brain (Tonnies and Trushina, 2017). ROS-induced mitochondrial oxidative stress promotes further destruction and dysfunction of mitochondria, thus forming a vicious cycle in the AD brain (Singh et al., 2019). DHYZ is composed of 15 kinds of herbs, which contain a variety of active ingredients including loganin, echinacoside, verbascoside, etc., which have antioxidant capacity and can inhibit the generation of ROS (Wang et al., 2015; Cheng et al., 2020; Vasincu et al., 2020). Our previous study showed that echinacoside could inhibit the accumulation of misfolded protein, including Aβ through improving endoplasmic reticulum stress (Dai et al., 2020). Consistent with those findings, our data in the present study revealed that ROS release increased and the activities of the respiratory chain complex I, II, III, and IV decreased dramatically in the brain of 2× Tg-AD mice. In light of the protective role against mitochondrial membrane destruction, DHYZ could depress the ROS overproduction and ameliorate the activity of the respiratory chain in mitochondria, thus blocking the vicious cycle of ROS release and mitochondrial dysfunction.

Choline is the precursor of acetylcholine which can activate acetylcholine receptors and further participate in the immune response of the CNS, and the imbalance of choline can lead to the occurrence of AD (Velazquez et al., 2019). The cholinergic hypothesis proposed that the pathogenesis of AD is closely related to the reduction of acetylcholine synthesis (Bartus et al., 1982). Wang et al. (2019) found that the levels of choline and acetylcholine in the hippocampus and cerebral cortex of APP/PS1 transgenic mice were significantly decreased. Ladner and Lee (1998) reported that the increase of acetylcholinesterase AChE in the brain tissue of AD patients can rapidly degrade acetylcholine ACh, thereby worsening the pathological state of AD patients. AChE is one of the key target enzymes in AD, which still represent the main pharmacotherapeutic approach in AD treatment. As metabolomics analysis confirmed in the present study, the content of phosphatidate was reduced in 2× Tg-AD mice. Phosphatidate is an important substrate for the synthesis of choline which can be converted into phosphatidylcholine which is the precursor of choline and acetylcholine (Lee and Ridgway, 2020). Our data presented here revealed that the levels of choline and acetylcholine in 2× Tg-AD mice were reduced, and these changes were reversed by DHYZ treatment *via* depressing the expression of AChE.

Niacin is the amide form of vitamin B3, including two forms of nicotinic acid and niacinamide which are closely related to neuronal development, survival, and death, and exhibits neuroprotective effects on neurodegenerative diseases, including AD, Parkinson’s disease, and Huntington’s diseases (Gasperi et al., 2019). As the precursors of NAD^+^, nicotinic acid and nicotinamide are more intimately involved in the salvage synthesis process of NAD^+^ (Martens et al., 2018). There is an obvious disturbance in nicotinate and nicotinamide metabolism in the mouse model and patients who suffered from AD (Spector, 1979; Fu et al., 2014; Rawji et al., 2020; Mousa and Mousa, 2021). PARP-1 plays a key role in AD pathogenesis and participates in multiple stress processes including DNA damage repair, inflammation, autophagy dysregulation, genomic stability maintenance, differentiation (Mao and Zhang, 2021). In the brains of AD patients, ROS overproduction leads to mitochondrial damage and the double-strand DNA breaks, resulting in the activation of PARP-1. It has been shown that PARP1 activity is increased in AD, and may exacerbate inflammatory response *via* NF-κB (Chiarugi and Moskowitz, 2003; Salech et al., 2017). The activated PARP-1 catalyzes the poly (ADP-ribosylation), which cleaves the NAD + and transfers the ADP-ribose moieties to the enzyme itself or to an acceptor protein to form branched polymers of ADP-ribose (Martire et al., 2016; Ryu et al., 2018). Previous studies have found that the reduction of nicotinic acid and niacinamide is closely related to the pathogenesis of AD (Mousa and Mousa, 2021). Yu et al. (2021) found that metabolites related to the metabolism of nicotinic acid and niacinamide decreased in drosophila overexpressing Aβ through the metabolomics analysis. After being given nicotinamide as a supplement, the mitochondrial defects and the behavioral disorders were rescued (Yu et al., 2021). NAD^+^ is a cofactor of the dehydrogenation of glyceraldehyde 3-phosphate in the glycolysis process (Baker et al., 2014). In 2× Tg-AD mice, the level of NAD^+^ decreases, which hinders the glycolysis process, and results in obstacles to the production of L-lactic acid which is transferred from astrocytes to neuron as fuel for the TCA cycle, a process known as the astrocyte-neuron lactate shuttle (ANLS) (Figure 10). In our experiment, DHYZ could depress the upregulation of PARP-1 in 2× Tg-AD mice, which was the potential target of DHYZ against AD. Furthermore, the content of urine nicotinic acid, niacinamide, and cerebral NAD^+^ decreased in 2× Tg-AD mice. This data is consistent with the upregulation of PARP-1 in the brain of 2× Tg-AD mice. Administration of DHYZ dramatically increased the content of nicotinic acid, nicotinamide, and NAD^+^ in 2× Tg-AD mice. Notably, as an inhibitor of PARP-1, nicotinamide may provide therapeutic benefits in AD by diminishing neuroinflammation, microglial activation, oxidative stress, and apoptosis (Turunc Bayrakdar et al., 2014; Salech et al., 2020). Our results presented here showed therapeutic potential for DHYZ against AD targeting PARP-1 and nicotinamide metabolism.

Glycolysis disturbance is another crucial event in AD pathogenesis (Fu and Jhamandas, 2014). With aging, the main risk factors for AD, have intensified, the utilization of glucose by the brain through glycolysis has also been significantly reduced (Yao et al., 2011; Ding et al., 2013). Lactic acid, the main product of glycolysis, is generated in astrocytes and transferred into neurons as substrate convert to pyruvic acid for energy production with the TCA cycle (Fu and Jhamandas, 2014; Sun et al., 2020). Reduced lactate content in the neurons of the hippocampus and cortex was observed in the APP/PS1 mouse model, which in turn was associated with an energy crisis, downregulated expression of long-term memory-related proteins, and resulted in memory deficits (Schousboe et al., 2003; Zhou et al., 2018). Increasing the content of lactic acid through drug intervention can reverse the cognitive impairment of AD mice (Lu et al., 2019). Our results are in line with a number of previous studies (Zhang M. et al., 2018; El Hayek et al., 2019), suggesting lactic acid levels are significantly reduced in 2× Tg-AD mice, implying that glycolysis is disrupted, which may interfere with neuronal energy metabolism. After the intervention of DHYZ, the content of lactic acid in 2× Tg-AD mice increased, which means that glycolysis disorders were ameliorated. Methylmalonic acid (MMA) is a dicarboxylic acid related to the metabolism of several amino acids and generated with ALDH3B2 in glycolysis (Kolker et al., 2003). Serot, et al. revealed that CSR MMA was significantly lower in aging subjects *vs.* younger ones, however, MMA showed a higher level in CSF of AD subjects as compared to healthy control (Joosten et al., 1997; Serot et al., 2005). Interestingly, our data showed a decreased urine MMA level in 2× Tg-AD mice which partly may be attributed to the effect of separation of the blood-brain barrier. Furthermore, our results showed that ALDH3B2, another potential target of DHYZ against AD, was specifically expressed in astrocytes, implying that ALDH3B2 may play a key role in aerobic glycolysis.

The brain is the organ with the highest metabolism and energy demand, which is up to 20% of whole-body energy in humans, and proper brain function produces large dynamic variations in energy metabolism through the TCA cycle coupled with OxPhos (Arnold et al., 2018). Due to this, energy metabolism impairment has been considered as both an early marker of AD pathology and a risk factor for AD, and the disruption of energy metabolism is regarded as a prominent feature, a fundamental component, and even a novel biomarker of AD pathogenesis (Weise et al., 2018; Butterfield and Halliwell, 2019; Perez Ortiz and Swerdlow, 2019). One of the main characteristics of AD is the severe region-specific decline of the cerebral metabolic rate for glucose (CMRglc). Furthermore, the decrease of CMRglc correlates with dementia severity in AD as the disease progression. Besides, the enzymes involved in the TCA cycle and OxPhos downregulate in the AD brain. By studying the cerebrospinal fluid (CSF) and plasma metabolism of AD patients, Van der Velpen et al. found that they exhibited disrupted core energy metabolism based on the TCA cycle, and the main intermediate metabolites involved, including cis-aconitic acid, citric acid, pyruvic acid, and phosphoenolpyruvic acid, were significantly increased (van der Velpen et al., 2019; Yan et al., 2020). Arnold et al. (2020) confirmed there were significant differences in the level of metabolites related to energy metabolism and mitochondrial function in the serum of AD and MCI patients by investigating serum metabolomics of females with APOE ε4 genotype. In the neuronal energy metabolism in CNS, the ANLS between astrocytes and neurons connects the glycolysis occurring in astrocytes with the TCA cycle in neurons. Then, the L-lactic acid is converted to pyruvic acid in the neuron. DAO level was significantly associated with the severity of the cognitive deficits in AD patients (Lin et al., 2017). DAO and amino acids can regulate the N-methyl-D-aspartate (NMDA) receptor function and DAO can catalyze the conversion of D-alanine to pyruvate in the peroxisome, which in turn enters the TCA cycle (Lin et al., 2017). At the same time, PDH oxidized pyruvic acid to form acetyl-CoA entering the TCA cycle. PDH may be inactivated through phosphorylation by PDHK1 which is upregulated by HIF1A. Our previous studies revealed that DHYZ could significantly ameliorate cerebral energy metabolism deficits (Ma et al., 2012; Huang et al., 2018). In our present study, ATP production and EC were declined in the brain tissue of 2× Tg-AD mice, and DHYZ could markedly ameliorate the disturbance of energy production. By increasing L-lactate levels, DHYZ promotes ANLS, which in turn increases neuronal energy metabolism. Furthermore, our data revealed that the levels of 4 key metabolites, including pyruvic acid, citric acid, succinic acid, and fumaric acid, were significantly recovered after DHYZ treatment, which means that DHYZ can ameliorate TCA cycle disorders. As a potential target of DHYZ against AD, DAO was upregulated in the brain of 2× Tg-AD mice after DHYZ treatment, which promoted the pyruvic acid supplement to enter TCA cycle. Furthermore, DHYZ could promote the TCA cycle by depressing HIF1A and PDHK1, and the phosphorylation of PDH to improve pyruvic acid entry into the TCA cycle.

Kyoto Encyclopedia of Genes and Genomes enrichment revealed that DHYZ had multiple protective efficacies upon the neurodegenerative diseases, including AD, Parkinson’s disease (PD), and Huntington’s disease (HD). In line with clinical applications of DHYZ for a long time, we found that therapeutic drug targets mostly concentrated in neurodegenerative diseases, such as AD, PD, and PD, and the majority of the targets were closely related to energy metabolism. KEGG and GO enrichment analysis of our current study revealed that DHYZ affected OxPhos with high –log_10_ (*p*-value). At the same time, NAFLD was also a key disease with KEGG enrichment of DHYZ targets. A reasonable explanation for this might be that AD and NAFLD share some common pathogenesis and mechanism related to abnormal lipid metabolism as demonstrated by many studies (de la Monte, 2017; Wang et al., 2020).

## Conclusion

Our study demonstrated that DHYZ improves cognitive performance in AD mice by targeting 4 key energy-related metabolic pathways. The regulation of the above metabolic pathways by DHYZ involves 5 key target genes and 14 differential metabolites. The central key to the regulation of 4 energy-related metabolic pathways by DHYZ is the protection of mitochondrial structure and function. Our data have implications for exploring the comprehensive mechanism of traditional Chinese herbal prescription in the treatment of AD.

## Data Availability Statement

The original contributions presented in the study are included in the article/Supplementary Material, further inquiries can be directed to the corresponding author/s.

## Ethics Statement

The animal study was reviewed and approved by Animal Care and Welfare Committee of Dongfang Hospital, Beijing University of Chinese Medicine, China.

## Author Contributions

TM designed the research and projected the experimental approach. GH, WZ, YD, HY, and DL performed the research and analyzed the data. GH, WZ, HY, DL, and TM wrote the manuscript. All authors contributed to the article and approved the final manuscript.

## Conflict of Interest

The authors declare that the research was conducted in the absence of any commercial or financial relationships that could be construed as a potential conflict of interest.

## Publisher’s Note

All claims expressed in this article are solely those of the authors and do not necessarily represent those of their affiliated organizations, or those of the publisher, the editors and the reviewers. Any product that may be evaluated in this article, or claim that may be made by its manufacturer, is not guaranteed or endorsed by the publisher.

## Footnotes

1
http://bionet.ncpsb.org/batman-tcm

2
http://www.swissadme.ch

3
https://old.tcmsp-e.com/tcmsp.php

4
https://pubchem.ncbi.nlm.nih.gov/

5
http://www.Swisstargetprediction.ch/

6
https://sea.bkslab.org/

7
https://www.genecards.org/

8
http://www.uniprot.org/

9
https://metascape.org/gp/index.html#/main/step1

10
https://string-db.org

11
https://www.metaboanalyst.ca/
