# Supplementary material for: Dihuang-Yinzi Alleviates Cognition Deficits via Targeting Energy-Related Metabolism in an Alzheimer Mouse Model as Demonstrated by Integration of Metabolomics and Network Pharmacology
Source: Front Aging Neurosci. 2022 Apr 1;14:873929. doi: 10.3389/fnagi.2022.873929 (PMC9011333; doi:10.3389/fnagi.2022.873929)
Supplement: Supplementary file 1 [file Table_1.DOCX]

**Supplementary TABLE 1** Active composite compounds in DHYZ

| No. | PubChem CID | Molecle Name | Molecle formula | OB(%) | DL | GI absorption | Herbs | Source Database |
| --- | --- | --- | --- | --- | --- | --- | --- | --- |
| 1 | 395120 | Diop | C_31_H_32_O_2_P_2_ | 43.59 | 0.39 | / | Morinda，Dogwood | TCMSP |
| 2 | 5363269 | Ethyl oleate (NF) | C_20_H_38_O_2_ | 32.40 | 0.19 | / | Morinda，Dogwood | TCMSP |
| 3 | 222284 | Beta-sitosterol | C_29_H_50_O | 36.91 | 0.75 | / | Morinda，Dogwood，Cistanche，Jujube，Ginger | TCMSP |
| 4 | 222284 | Sitosterol | C_29_H_50_O | 36.91 | 0.75 | / | Morinda，Dogwood，Rehmannia，Aconite，Mint | TCMSP |
| 5 | 5280794 | Stigmasterol | C_29_H_48_O | 43.83 | 0.76 | / | Rehmannia，Dogwood，Jujube，Ginger | TCMSP |
| 6 | 457801 | Poriferast-5-en-3beta-ol | C_29_H_50_O | 36.91 | 0.75 | / | Dogwood，Ginger | TCMSP |
| 7 | 5280343 | Quercetin | C_15_H_10_O_7_ | 46.43 | 0.28 | / | Cistanche，Jujube | TCMSP |
| 8 | 5281800 | *Verbascoside | C_29_H_36_O_15_ | 2.94 | 0.62 | / | Rehmannia | TCMSP |
| 9 | 5282184 | Mandenol | C_20_H_36_O_2_ | 42.00 | 0.19 | / | Dogwood | TCMSP |
| 10 | 5367460 | Ethyl linolenate | C_20_H_34_O_2_ | 46.10 | 0.2 | / | Dogwood | TCMSP |
| 11 | N/A | 2,6,10,14,18-pentamethylicosa-2,6,10,14,18-pentaene | C_25_H_42_ | 33.4 | 0.24 | / | Dogwood | TCMSP |
| 12 | 442735 | Cornudentanone | C_22_H_34_O_5_ | 39.66 | 0.33 | / | Dogwood | TCMSP |
| 13 | 5318214 | Hydroxygenkwanin | C_16_H_12_O_6_ | 36.47 | 0.27 | / | Dogwood | TCMSP |
| 14 | 259991 | Telocinobufagin | C_24_H_34_O_5_ | 69.99 | 0.79 | / | Dogwood | TCMSP |
| 15 | 72340 | Tetrahydroalstonine | C_21_H_24_N_2_O_3_ | 32.42 | 0.81 | / | Dogwood | TCMSP |
| 16 | 87691 | *Loganin | C_17_H_26_O_10_ | 5.9 | 0.44 | / | Dogwood | TCMSP |
| 17 | 444899 | arachidonate | C_20_H_32_O_2_ | 45.57 | 0.2 | / | Cistanche | TCMSP |
| 18 | 132350840 | suchilactone | C_21_H_20_O_6_ | 57.52 | 0.56 | / | Cistanche | TCMSP |
| 19 | 443028 | Yangambin | C_24_H_30_O_8_ | 57.53 | 0.81 | / | Cistanche | TCMSP |
| 20 | 72341 | Marckine | C_29_H_37_N_3_O_3_ | 37.05 | 0.69 | / | Cistanche | TCMSP |
| 21 | 5281771 | echinacoside_qt | C_35_H_46_O_20_ | 2.94 | 0.62 | / | Cistanche | TCMSP |
| 22 | N/A | Alizarin-2-methylether | C_15_H_10_O_4_ | 32.81 | 0.21 | / | Morinda | TCMSP |
| 23 | 16203753 | 2-hydroxy-1,5-dimethoxy-6-(methoxymethyl)-9,10-anthraquinone | C_18_H_16_O_6_ | 95.85 | 0.37 | / | Morinda | TCMSP |
| 24 | N/A | 1,5,7-trihydroxy-6-methoxy-2-methoxymethylanthracenequinone | N/A | 80.42 | 0.38 | / | Morinda | TCMSP |
| 25 | 11674044 | 1,6-dihydroxy-5-methoxy-2-(methoxymethyl)-9,10-anthraquinone | C_17_H_14_O_6_ | 104.54 | 0.34 | / | Morinda | TCMSP |
| 26 | N/A | 1-hydroxy-6-hydroxymethylanthracenequinone | C_15_H_10_O_4_ | 81.77 | 0.21 | / | Morinda | TCMSP |
| 27 | N/A | 2-hydroxy-1,8-dimethoxy-7-methoxymethylanthracenequinone | N/A | 112.3 | 0.37 | / | Morinda | TCMSP |
| 28 | N/A | (2R,3S)-(+)-3',5-Dihydroxy-4 ,7-dimethoxydihydroflavonol | C_17_H_16_O_7_ | 77.24 | 0.33 | / | Morinda | TCMSP |
| 29 | N/A | 3beta,20(R),5-alkenyl-stigmastol | C_29_H_50_O | 36.91 | 0.75 | / | Morinda | TCMSP |
| 30 | N/A | 3beta-24S(R)-butyl-5-alkenyl-cholestol | N/A | 35.35 | 0.82 | / | Morinda | TCMSP |
| 31 | 5459018 | americanin A | C_18_H_16_O_6_ | 46.71 | 0.35 | / | Morinda | TCMSP |
| 32 | N/A | isoprincepin | N/A | 49.12 | 0.77 | / | Morinda | TCMSP |
| 33 | 442531 | Ohioensin-A | C_23_H_16_O_5_ | 38.13 | 0.76 | / | Morinda | TCMSP |
| 34 | 5282805 | 11,14-eicosadienoic acid | C_20_H_36_O_2_ | 39.99 | 0.2 | / | Aconite | TCMSP |
| 35 | N/A | Delphin_qt | C_27_H_31_ClO_17_ | 57.76 | 0.28 | / | Aconite | TCMSP |
| 36 | 906525 | Deltoin | N/A | 46.69 | 0.37 | / | Aconite | TCMSP |
| 37 | 21679042 | Deoxyandrographolide | C_20_H_30_O_4_ | 56.3 | 0.31 | / | Aconite | TCMSP |
| 38 | 100633 | Karanjin | C_18_H_12_O_4_ | 69.56 | 0.34 | / | Aconite | TCMSP |
| 39 | 70698023 | Longikaurin A | C_20_H_28_O_5_ | 47.72 | 0.53 | / | Schisandra | TCMSP |
| 40 | 285342 | Deoxyharringtonine | C_28_H_37_NO_8_ | 39.27 | 0.81 | / | Schisandra | TCMSP |
| 41 | 91864462 | Angeloylgomisin O | N/A | 31.97 | 0.85 | / | Schisandra | TCMSP |
| 42 | 5318785 | Schizandrer B | C_28_H_34_O_9_ | 30.71 | 0.83 | / | Schisandra | TCMSP |
| 43 | 3001662 | Gomisin-A | C_23_H_28_O_7_ | 30.69 | 0.78 | / | Schisandra | TCMSP |
| 44 | 11516888 | Gomisin R | N/A | 34.84 | 0.86 | / | Schisandra | TCMSP |
| 45 | 443027 | Wuweizisu C | C_22_H_24_O_6_ | 46.27 | 0.84 | / | Schisandra | TCMSP |
| 46 | 129716399 | 8-Isopentenyl-kaempferol | C_20_H_18_O_6_ | 38.04 | 0.39 | / | Calamus | TCMSP |
| 47 | N/A | (1R,3aS,4R,6aS)-1,4-bis(3,4-dimethoxyphenyl)-1,3,3a,4,6,6a-hexahydrofuro[4,3-c]furan | C_22_H_26_O_6_ | 52.35 | 0.62 | / | Calamus | TCMSP |
| 48 | 92110 | Cycloartenol | C_30_H50O | 38.69 | 0.78 | / | Calamus | TCMSP |
| 49 | 5280863 | kaempferol | C_15_H_10_O_6_ | 41.88 | 0.24 | / | Calamus | TCMSP |
| 50 | 10743008 | (2R)-2-[(3S,5R,10S,13R,14R,16R,17R)-3,16-dihydroxy-4,4,10,13,14-pentamethyl-2,3,5,6,12,15,16,17-octahydro-1H-cyclopenta[a]phenanthren-17-yl]-6-methylhept-5-enoic acid | C_30_H_46_O_4_ | 30.93 | 0.81 | / | Poria | TCMSP |
| 51 | 12309443 | trametenolic acid | C_30_H_48_O_3_ | 38.71 | 0.8 | / | Poria | TCMSP |
| 52 | 10181133 | Cerevisterol | C_28_H_46_O_3_ | 37.96 | 0.77 | / | Poria | TCMSP |
| 53 | N/A | ergosta-7,22E-dien-3beta-ol | C_28_H_46_O | 43.51 | 0.72 | / | Poria | TCMSP |
| 54 | 5351516 | Ergosterol peroxide | N/A | 40.36 | 0.81 | / | Poria | TCMSP |
| 55 | 73299 | hederagenin | C_30_H_48_O_4_ | 36.91 | 0.75 | / | Poria | TCMSP |
| 56 | N/A | 6-methylgingediacetate2 | N/A | 48.73 | 0.32 | / | Ginger | TCMSP |
| 57 | 98455 | stepharine | C_18_H_19_NO_3_ | 31.55 | 0.33 | / | Jujube | TCMSP |
| 58 | N/A | zizyphus saponin I_qt | N/A | 32.69 | 0.62 | / | Jujube | TCMSP |
| 59 | 5281707 | coumestrol | C_15_H_8_O_5_ | 32.49 | 0.34 | / | Jujube | TCMSP |
| 60 | N/A | Daechuine S7 | N/A | 44.82 | 0.83 | / | Jujube | TCMSP |
| 61 | 101635353 | Jujubasaponin V_qt | N/A | 36.99 | 0.63 | / | Jujube | TCMSP |
| 62 | 6443026 | Mauritine D | N/A | 89.13 | 0.45 | / | Jujube | TCMSP |
| 63 | 2353 | berberine | C_20_H_19_NO_8_S | 36.86 | 0.78 | / | Jujube | TCMSP |
| 64 | 160487 | (S)-Coclaurine | C_17_H_19_NO_3_ | 42.35 | 0.24 | / | Jujube | TCMSP |
| 65 | 64971 | Mairin | C_30_H_48_O_3_ | 55.38 | 0.78 | / | Jujube | TCMSP |
| 66 | 101650325 | Ruvoside_qt | C_30_H_46_O_9_ | 36.12 | 0.76 | / | Jujube | TCMSP |
| 67 | 9064 | (+)-catechin | C_15_H_14_O_6_ | 54.83 | 0.24 | / | Jujube | TCMSP |
| 68 | 6917970 | Stepholidine | C_19_H_21_NO_4_ | 33.11 | 0.54 | / | Jujube | TCMSP |
| 69 | 10146 | Nuciferin | C_19_H_21_NO_2_ | 34.43 | 0.4 | / | Jujube | TCMSP |
| 70 | 4970 | Fumarine | C_20_H_19_NO_5_ | 59.26 | 0.83 | / | Jujube | TCMSP |
| 71 | 5280489 | beta-carotene | C_40_H_56_ | 37.18 | 0.58 | / | Jujube | TCMSP |
| 72 | 73160 | (-)-catechin | C_15_H_14_O_6_ | 49.68 | 0.24 | / | Jujube | TCMSP |
| 73 | 5280442 | acacetin | C_16_H_12_O_5_ | 34.97 | 0.24 | / | Mint | TCMSP |
| 74 | 5281612 | Diosmetin | C_16_H_12_O_6_ | 31.14 | 0.27 | / | Mint | TCMSP |
| 75 | 932 | naringenin | C_15_H_12_O_5_ | 59.29 | 0.21 | / | Mint | TCMSP |
| 76 | 10207 | aloe-emodin | C_15_H_10_O_5_ | 83.38 | 0.24 | / | Mint | TCMSP |
| 77 | 440735 | eriodictyol | C_15_H_12_O_6_ | 71.79 | 0.24 | / | Mint | TCMSP |
| 78 | 5281617 | Genkwanin | C_16_H_12_O_5_ | 37.13 | 0.24 | / | Mint | TCMSP |
| 79 | 5280445 | luteolin | C_15_H_10_O_6_ | 36.16 | 0.25 | / | Mint | TCMSP |
| 80 | 637511 | Cinnamic Aldehyde | C_9_H_8_O | / | A | High | Cinnamon | BATMAN |
| 81 | 323 | Coumarin | C_9_H_6_O_2_ | / | A | High | Cinnamon | BATMAN |
| 82 | 442792 | Melilotocarpan A | C_17_H_16_O_5_ | / | A | High | Cinnamon | BATMAN |
| 83 | 5705112 | Cinnamyl Benzoate | C_16_H_14_O_2_ | / | A | High | Cinnamon | BATMAN |
| 84 | 5315892 | Cinnamic Alcohol | C_9_H_10_O | / | A | High | Cinnamon | BATMAN |
| 85 | 637563 | Anethole | C_10_H_12_O | / | A | High | Cinnamon | BATMAN |
| 86 | 444539 | Trans-Cinnamic Acid | C_9_H_8_O_2_ | / | A | High | Cinnamon | BATMAN |
| 87 | 637758 | Ethylcinnamate | C_11_H_12_O_2_ | / | A | High | Cinnamon | BATMAN |
| 88 | 72 | Protocatechuic Acid | C_7_H_6_O_4_ | / | A | High | Cinnamon | BATMAN |
| 89 | 5282110 | Cinnamyl Acetate | C_11_H_12_O_2_ | / | A | High | Cinnamon | BATMAN |
| 90 | 10423984 | Denbinobin | C_16_H_12_O_5_ | / | A | High | Dendrobium | BATMAN |
| 91 | 168974 | Shihunin | C_12_H_13_NO_2_ | / | A | High | Dendrobium | BATMAN |
| 92 | 342801 | Dentatin | C_20_H_22_O_4_ | / | A | High | Dendrobium | BATMAN |
| 93 | 5316534 | Dendrolasin | C_15_H_22_O | / | A | High | Dendrobium | BATMAN |
| 94 | 26305 | Nodakenetin | C_14_H_14_O_4_ | / | A | High | Dendrobium | BATMAN |
| 95 | 14826840 | Ophiopogonone B | C_18_H_16_O_5_ | / | A | High | Ophiopogon | BATMAN |
| 96 | 181686 | Orchinol | C_16_H_16_O_3_ | / | A | High | Ophiopogon | BATMAN |
| 97 | 10871974 | Ophiopogonanone C | C_19_H_16_O_7_ | / | A | High | Ophiopogon | BATMAN |
| 98 | 5316797 | Ophiopogonanone E | C_19_H_20_O_7_ | / | A | High | Ophiopogon | BATMAN |
| 99 | 5280537 | N-Trans-Feruloyltyramine | C_18_H_19_NO_4_ | / | A | High | Ophiopogon | BATMAN |
| 100 | 10087732 | Ophiopogonone A | C_18_H_14_O_6_ | / | A | High | Ophiopogon | BATMAN |
| 101 | 9996586 | Ophiopogonanone A | C_18_H_16_O_6_ | / | A | High | Ophiopogon | BATMAN |
| 102 | 99474 | Diosgenin | C_27_H_42_O_3_ | / | A | High | Ophiopogon | BATMAN |
| 103 | 441893 | Ruscogenin | C_27_H_42_O_4_ | / | A | High | Ophiopogon | BATMAN |
| 104 | 5316766 | 1,6-Dihydroxy-3,7-Dimethoxyxanthone | C_15_H_12_O_6_ | / | A | High | Polygala | BATMAN |
| 105 | 23618202 | S-(2-Carboxyethyl)-L-Cysteine | C_6_H_11_NO_4_S | / | A | High | Polygala | BATMAN |
| 106 | 442290 | 1-Peroxyferolide | C_17_H_22_O_7_ | / | A | High | Polygala | BATMAN |
| 107 | N/A | 2-Peroxyferolide | N/A | / | A | High | Polygala | BATMAN |
| 108 | 227830 | Tenulin | C_17_H_22_O_5_ | / | A | High | Polygala | BATMAN |
| 109 | 5316837 | 1,6-Dihydroxy-3,5,7-Trimethoxyxanthone | C_16_H_14_O_7_ | / | A | High | Polygala | BATMAN |
| 110 | 148724 | 5,6,7-Trimethoxycoumarin | C_12_H_12_O_5_ | / | A | High | Polygala | BATMAN |
| 111 | 5281618 | Geraldone | C_16_H_12_O_5_ | / | A | High | Polygala | BATMAN |
| 112 | 5321809 | Tenuifoliside D | C_18_H_24_O_9_ | / | A | High | Polygala | BATMAN |
| 113 | 64961 | Norharman | C_11_H_8_N_2_ | / | A | High | Polygala | BATMAN |
| 114 | 160179 | Perlolyrine | C_16_H_12_N_2_O_2_ | / | A | High | Polygala | BATMAN |
| 115 | 64696 | Norhyoscyamine | C_16_H_21_NO_3_ | / | A | High | Polygala | BATMAN |
| 116 | 21588226 | Tenuifolin | C_36_H_56_O_12_ | / | A | High | Polygala | BATMAN |
| 117 | 5280953 | Harmine | C_13_H_12_N_2_O | / | A | High | Polygala | BATMAN |
| 118 | 5281404 | Harman | C_12_H_10_N_2_ | / | A | High | Polygala | BATMAN |
| 119 | 5488808 | 1-Hydroxy-3,7-Dimethoxyxanthone | C_15_H_12_O_5_ | / | A | High | Polygala | BATMAN |
| 120 | 145865806 | Tenuifoliose A | _C62_H_76_O_35_ | / | A | High | Polygala | BATMAN |
| 121 | 11972435 | Polygalaxanthone Iv | C_27_H_32_O_15_ | / | A | High | Polygala | BATMAN |
| 122 | 5316765 | 1,3-Dihydroxy-4,5-Dimethoxyxanthone | C_15_H_12_O_6_ | / | A | High | Polygala | BATMAN |
| 123 | 5320290 | Onjixanthone I | C_16_H_14_O_6_ | / | A | High | Polygala | BATMAN |

Note: In this table, the first 79 active ingredients of herbs are obtained through the TCMSP database according to the standards of OB≥30% and DL≥0.18, "*" means that the active ingredients are added through the "Pharmacopeia of the People's Republic of China"; last 44 the active ingredients of herbs are obtained through the BATMAN database according to the GI absorption as “High” and the DL as "A" in the SwissADME; "A" means that the ingredient meets the 3 conditions of drug-like properties in the SwissADME database, "/" indicates that the active ingredient has not been added in accordance with this standard.
