# Supplementary material for: Dihuang-Yinzi Alleviates Cognition Deficits via Targeting Energy-Related Metabolism in an Alzheimer Mouse Model as Demonstrated by Integration of Metabolomics and Network Pharmacology
Source: Front Aging Neurosci. 2022 Apr 1;14:873929. doi: 10.3389/fnagi.2022.873929 (PMC9011333; doi:10.3389/fnagi.2022.873929)
Supplement: Supplementary file 2 [file Table_2.DOCX]

**Supplementary TABLE 2** 192 potential targeted of DHYZ against AD.

| No. | Gene name | Protein |
| --- | --- | --- |
| 1 | ADH1C | Alcohol dehydrogenase 1C |
| 2 | PTGS1 | Prostaglandin G/H synthase 1 |
| 3 | PTGS2 | Prostaglandin G/H synthase 2 |
| 4 | ADRA2A | Alpha-2A adrenergic receptor |
| 5 | SLC6A2 | Sodium-dependent noradrenaline transporter |
| 6 | SLC6A3 | Sodium-dependent dopamine transporter |
| 7 | AKR1B1 | Aldo-keto reductase family 1 member B1 |
| 8 | MAOB | Amine oxidase [flavin-containing] B |
| 9 | MAOA | Amine oxidase [flavin-containing] A |
| 10 | HTR2A | 5-hydroxytryptamine receptor 2A |
| 11 | ADRA1A | Alpha-1D adrenergic receptor |
| 12 | MMP2 | 72 kDa type IV collagenase |
| 13 | APP | Amyloid-beta precursor protein |
| 14 | DRD1 | D(1A) dopamine receptor |
| 15 | GABRA2 | Gamma-aminobutyric acid receptor subunit alpha-2 |
| 16 | SLC6A4 | Sodium-dependent serotonin transporter |
| 17 | BCL2 | Apoptosis regulator Bcl-2 |
| 18 | JUN | Transcription factor AP-1 |
| 19 | CASP3 | Caspase-3 |
| 20 | NOS2 | Nitric oxide synthase |
| 21 | ACHE | Acetylcholinesterase |
| 22 | ADORA2A | Adenosine receptor A2a |
| 23 | ADA | Adenosine deaminase |
| 24 | SLC29A1 | Equilibrative nucleoside transporter 1 |
| 25 | MMP3 | Stromelysin-1 |
| 26 | NOS3 | Nitric oxide synthase, endothelial |
| 27 | RELA | Nanos homolog 3 |
| 28 | EGFR | Epidermal growth factor receptor |
| 29 | AKT1 | RAC-alpha serine/threonine-protein kinase |
| 30 | FOS | Proto-oncogene c-Fos |
| 31 | EIF6 | Eukaryotic translation initiation factor 6 |
| 32 | MMP9 | Matrix metalloproteinase-9 |
| 33 | MAPK1 | Mitogen-activated protein kinase 1 |
| 34 | IL10 | Interleukin-10 |
| 35 | IL6 | Interleukin-6 |
| 36 | TP53 | Cellular tumor antigen p53 |
| 37 | XDH | Xanthine dehydrogenase/oxidase |
| 38 | SOD1 | Superoxide dismutase [Cu-Zn] |
| 39 | HIF1A | Hypoxia-inducible factor 1-alpha |
| 40 | HMOX1 | Heme oxygenase 1 |
| 41 | CYP1A2 | Cytochrome P450 1A2 |
| 42 | MYC | Myc proto-oncogene protein |
| 43 | PRKCB | Protein kinase C beta type |
| 44 | HSPB1 | Heat shock protein beta-1 |
| 45 | CCNB1 | G2/mitotic-specific cyclin-B1 |
| 46 | COL1A1 | Collagen alpha-1 |
| 47 | IFNG | Interferon gamma |
| 48 | MPO | Myeloperoxidase |
| 49 | NCF1 | Neutrophil cytosol factor 1 |
| 50 | GSTP1 | Glutathione S-transferase P |
| 51 | NFE2L2 | Nuclear factor erythroid 2-related factor 2 |
| 52 | NQO1 | NAD(P)H dehydrogenase [quinone] 1 |
| 53 | INSR | Insulin receptor |
| 54 | PPARA | Peroxisome proliferator-activated receptor alpha |
| 55 | PPARD | Peroxisome proliferator-activated receptor delta |
| 56 | HSF1 | Heat shock factor protein 1 |
| 57 | CHUK | Inhibitor of nuclear factor kappa-B kinase subunit alpha |
| 58 | IGF2 | Insulin-like growth factor II |
| 59 | GSK3B | Glycogen synthase kinase-3 beta |
| 60 | CCNA2 | Cyclin-A2 |
| 61 | ADH1A | Alcohol dehydrogenase 1A |
| 62 | ADH1B | All-trans-retinol dehydrogenase |
| 63 | CHRNA4 | Neuronal acetylcholine receptor subunit alpha-4 |
| 64 | TLR4 | Toll-like receptor 4 |
| 65 | HDAC2 | Histone deacetylase 2 |
| 66 | ADH4 | All-trans-retinol dehydrogenase |
| 67 | PARP1 | Poly [ADP-ribose] polymerase 1 |
| 68 | DAO | D-amino-acid oxidase |
| 69 | SRC | Proto-oncogene tyrosine-protein kinase Src |
| 70 | MET | Hepatocyte growth factor receptor |
| 71 | CYP19A1 | Aromatase |
| 72 | CDK1 | Cyclin-dependent kinase 1 |
| 73 | CDK5 | Cyclin-dependent-like kinase 5 |
| 74 | EGLN1 | Egl nine homolog 1 |
| 75 | ERN1 | Serine/threonine-protein kinase/endoribonuclease IRE1 |
| 76 | CDK2 | Cyclin-dependent kinase 2 |
| 77 | BACE1 | Beta-secretase 1 |
| 78 | ABL1 | Tyrosine-protein kinase ABL1 |
| 79 | MTOR | Serine/threonine-protein kinase mTOR |
| 80 | PIK3CA | Phosphatidylinositol 4,5-bisphosphate 3-kinase catalytic subunit alpha isoform |
| 81 | MAPK8 | Mitogen-activated protein kinase 8 |
| 82 | HDAC4 | Histone deacetylase 4 |
| 83 | PDK1 | 3-phosphoinositide-dependent protein kinase 1 |
| 84 | VCP | Transitional endoplasmic reticulum ATPase |
| 85 | SLC18A3 | Vesicular acetylcholine transporter |
| 86 | MAPK9 | Mitogen-activated protein kinase 9 |
| 87 | CHRNA3 | Neuronal acetylcholine receptor subunit alpha-3 |
| 88 | DRD2 | D(2) dopamine receptor |
| 89 | CYP11B2 | Cytochrome P450 11B2 |
| 90 | CNR1 | Cannabinoid receptor 1 |
| 91 | P2RY1 | P2Y purinoceptor 1 |
| 92 | CYP11B1 | Cytochrome P450 11B1 |
| 93 | IDO1 | Indoleamine 2,3-dioxygenase 1 |
| 94 | GSR | Glutathione reductase |
| 95 | SIGMAR1 | Sigma non-opioid intracellular receptor 1 |
| 96 | ALOX5 | Polyunsaturated fatty acid 5-lipoxygenase |
| 97 | MAPT | Microtubule-associated protein tau |
| 98 | CAPN2 | Calpain-2 catalytic subunit |
| 99 | AKR1C1 | Aldo-keto reductase family 1 member C1 |
| 100 | KMO | Kynurenine 3-monooxygenase |
| 101 | PDGFRA | Platelet-derived growth factor receptor alpha |
| 102 | ALDH2 | Aldehyde dehydrogenase, mitochondrial |
| 103 | LDHA | L-lactate dehydrogenase A chain |
| 104 | COMT | Catechol O-methyltransferase |
| 105 | ALDH1A1 | Aldehyde dehydrogenase 1 family member A1 |
| 106 | ALDH3B2 | Aldehyde dehydrogenase 3 family member B2 |
| 107 | LRRK2 | Leucine-rich repeat serine/threonine-protein kinase 2 |
| 108 | HRH3 | Histamine H3 receptor |
| 109 | PTK2B | Protein-tyrosine kinase 2-beta |
| 110 | JAK2 | Tyrosine-protein kinase JAK2 |
| 111 | GRM4 | Metabotropic glutamate receptor 4 |
| 112 | GAPDH | Glyceraldehyde-3-phosphate dehydrogenase |
| 113 | CALM1 | Calmodulin-1 |
| 114 | HKDC1 | Hexokinase HKDC1 |
| 115 | DRD4 | D(4) dopamine receptor |
| 116 | G6PD | Glucose-6-phosphate 1-dehydrogenase |
| 117 | SIRT1 | NAD-dependent protein deacetylase sirtuin-1 |
| 118 | NOX4 | NADPH oxidase 4 |
| 119 | SPHK1 | Sphingosine kinase 1 |
| 120 | ESRRB | Steroid hormone receptor ERR2 |
| 121 | DRD3 | D(3)dopamine receptor |
| 122 | MMP14 | Matrix metalloproteinase-14 |
| 123 | TRAP1 | Heat shock protein 75 kDa, mitochondrial |
| 124 | AVPR1A | Vasopressin V1a receptor |
| 125 | NR1H4 | Bile acid receptor |
| 126 | AKT2 | RAC-beta serine/threonine-protein kinase |
| 127 | IL6ST | Interleukin-6 receptor subunit beta |
| 128 | PYGL | Glycogen phosphorylase |
| 129 | GYS1 | Glycogen [starch] synthase |
| 130 | P4HB | Protein disulfide-isomerase |
| 131 | GLO1 | Lactoylglutathione lyase |
| 132 | DAPK1 | Death-associated protein kinase 1 |
| 133 | APEX1 | DNA-(apurinic or apyrimidinic site) endonuclease |
| 134 | MDH1 | Malate dehydrogenase |
| 135 | GCLC | Glutamate--cysteine ligase catalytic subunit |
| 136 | ARG2 | Arginase-2 |
| 137 | GFPT1 | Glutamine--fructose-6-phosphate aminotransferase [isomerizing] 1 |
| 138 | GRIK5 | Glutamate receptor ionotropic, kainate 5 |
| 139 | ARG1 | Arginase-1 |
| 140 | SLC1A1 | Excitatory amino acid transporter 3 |
| 141 | HSPA1A | Heat shock 70 kDa protein 1A |
| 142 | QDPR | Dihydropteridine reductase |
| 143 | CAMK2A | Calcium/calmodulin-dependent protein kinase type II subunit alpha |
| 144 | SLC22A3 | Solute carrier family 22 member 3 |
| 145 | EP300 | Histone acetyltransferase p300 |
| 146 | NDUFA4 | Cytochrome c oxidase subunit NDUFA4 |
| 147 | NDUFA1 | NADH dehydrogenase [ubiquinone] 1 alpha subcomplex subunit 1 |
| 148 | NDUFA2 | NADH dehydrogenase [ubiquinone] 1 alpha subcomplex subunit 2 |
| 149 | NDUFA3 | NADH dehydrogenase [ubiquinone] 1 alpha subcomplex subunit 3 |
| 150 | NDUFA5 | NADH dehydrogenase [ubiquinone] 1 alpha subcomplex subunit 5 |
| 151 | NDUFA6 | NADH dehydrogenase [ubiquinone] 1 alpha subcomplex subunit 6 |
| 152 | NDUFA8 | NADH dehydrogenase [ubiquinone] 1 alpha subcomplex subunit 8 |
| 153 | NDUFA9 | NADH dehydrogenase [ubiquinone] 1 alpha subcomplex subunit 9 |
| 154 | NDUFA10 | NADH dehydrogenase [ubiquinone] 1 alpha subcomplex subunit 10 |
| 155 | NDUFA11 | NADH dehydrogenase [ubiquinone] 1 alpha subcomplex subunit 11 |
| 156 | NDUFA12 | NADH dehydrogenase [ubiquinone] 1 alpha subcomplex subunit 12 |
| 157 | NDUFA13 | NADH dehydrogenase [ubiquinone] 1 alpha subcomplex subunit 13 |
| 158 | NDUFB2 | NADH dehydrogenase [ubiquinone] 1 beta subcomplex subunit 2 |
| 159 | NDUFB3 | NADH dehydrogenase [ubiquinone] 1 beta subcomplex subunit 3 |
| 160 | NDUFB4 | NADH dehydrogenase [ubiquinone] 1 beta subcomplex subunit 4 |
| 161 | NDUFB5 | NADH dehydrogenase [ubiquinone] 1 beta subcomplex subunit 5 |
| 162 | NDUFB6 | NADH dehydrogenase [ubiquinone] 1 beta subcomplex subunit 6 |
| 163 | NDUFB7 | NADH dehydrogenase [ubiquinone] 1 beta subcomplex subunit 7 |
| 164 | NDUFB8 | NADH dehydrogenase [ubiquinone] 1 beta subcomplex subunit 8 |
| 165 | NDUFB9 | NADH dehydrogenase [ubiquinone] 1 beta subcomplex subunit 9 |
| 166 | NDUFB10 | NADH dehydrogenase [ubiquinone] 1 beta subcomplex subunit 10 |
| 167 | NDUFB11 | NADH dehydrogenase [ubiquinone] 1 beta subcomplex subunit 11 |
| 168 | NDUFC2 | NADH dehydrogenase [ubiquinone] 1 subunit C2 |
| 169 | NDUFS1 | NADH-ubiquinone oxidoreductase 75 kDa subunit |
| 170 | NDUFS2 | NADH dehydrogenase [ubiquinone] iron-sulfur protein 2 |
| 171 | NDUFS3 | NADH dehydrogenase [ubiquinone] iron-sulfur protein 3 |
| 172 | NDUFS4 | NADH dehydrogenase [ubiquinone] iron-sulfur protein 4 |
| 173 | NDUFS5 | NADH dehydrogenase [ubiquinone] iron-sulfur protein 5 |
| 174 | NDUFS6 | NADH dehydrogenase [ubiquinone] iron-sulfur protein 6 |
| 175 | NDUFS7 | NADH dehydrogenase [ubiquinone] iron-sulfur protein 7 |
| 176 | NDUFS8 | NADH dehydrogenase [ubiquinone] iron-sulfur protein 8 |
| 177 | NDUFV1 | NADH dehydrogenase [ubiquinone] flavoprotein 1 |
| 178 | NDUFV2 | NADH dehydrogenase [ubiquinone] flavoprotein 2 |
| 179 | NDUFV3 | NADH dehydrogenase [ubiquinone] flavoprotein 3 |
| 180 | ABCC2 | ATP-binding cassette sub-family C member 2 |
| 181 | AKR1C4 | Aldo-keto reductase family 1 member C4 |
| 182 | AADAT | Kynurenine/alpha-aminoadipate aminotransferase |
| 183 | CAT | Catalase |
| 184 | MAPK3 | Mitogen-activated protein kinase 3 |
| 185 | BAD | Bcl2-associated agonist of cell death |
| 186 | ADIPOQ | Adiponectin |
| 187 | GOT1 | Aspartate aminotransferase |
| 188 | ABAT | 4-aminobutyrate aminotransferase |
| 189 | PCNA | Proliferating cell nuclear antigen |
| 190 | PRKCD | Protein kinase C delta type |
| 191 | MCL1 | Induced myeloid leukemia cell differentiation protein Mcl-1 |
| 192 | IL4 | Interleukin-4 |
