# Supplementary material for: Dihuang-Yinzi Alleviates Cognition Deficits via Targeting Energy-Related Metabolism in an Alzheimer Mouse Model as Demonstrated by Integration of Metabolomics and Network Pharmacology
Source: Front Aging Neurosci. 2022 Apr 1;14:873929. doi: 10.3389/fnagi.2022.873929 (PMC9011333; doi:10.3389/fnagi.2022.873929)
Supplement: Supplementary file 3 [file Table_3.DOCX]

**Supplementary TABLE 3** The construction of the protein interaction network

| No | Degree | Gene | Closeness centrality | Betweenness centrality | Topological coefficient |
| --- | --- | --- | --- | --- | --- |
| 1 | 68 | NDUFA12 | 0.8834 | 0.0182 | 0.7177 |
| 2 | 67 | NDUFS1 | 0.8715 | 0.0343 | 0.6304 |
| 3 | 67 | GAPDH | 0.8715 | 0.0481 | 0.6085 |
| 4 | 66 | IL10 | 0.8571 | 0.0794 | 0.5806 |
| 5 | 65 | AKT1 | 0.8549 | 0.0304 | 0.7652 |
| 6 | 65 | SIRT1 | 0.8549 | 0.0753 | 0.6515 |
| 7 | 65 | HIF1A | 0.8549 | 0.0739 | 0.7257 |
| 8 | 64 | NOS3 | 0.8493 | 0.045 | 0.7226 |
| 9 | 64 | HSPA1A | 0.8493 | 0.0548 | 0.4266 |
| 10 | 64 | PARP1 | 0.8493 | 0.0347 | 0.7419 |
| 11 | 63 | ARG1 | 0.8572 | 0.0287 | 0.626 |
| 12 | 62 | LDHA | 0.8310 | 0.0277 | 0.4899 |
| 13 | 62 | ACHE | 0.8310 | 0.0568 | 0.4736 |
| 14 | 62 | ALDH3B2 | 0.8310 | 0.0587 | 0.4517 |
| 15 | 61 | DAO | 0.7826 | 0.0256 | 0.594 |
| 16 | 60 | CASP3 | 0.7301 | 0.0216 | 0.6966 |
| 17 | 60 | MAPK8 | 0.7301 | 0.0353 | 0.409 |
| 18 | 60 | TP53 | 0.7301 | 0.0175 | 0.4202 |
| 19 | 60 | JUN | 0.7301 | 0.0617 | 0.5891 |
| 20 | 60 | MAPK3 | 0.7301 | 0.0668 | 0.577 |
| 21 | 60 | PTGS2 | 0.7301 | 0.039 | 0.6126 |
| 22 | 60 | MAPK1 | 0.7301 | 0.0691 | 0.4864 |
| 23 | 59 | MMP9 | 0.7610 | 0.023 | 0.641 |
| 24 | 59 | IL6 | 0.7610 | 0.0473 | 0.6774 |
| 25 | 59 | FOS | 0.7610 | 0.0628 | 0.7737 |
| 26 | 59 | NDUFA11 | 0.7610 | 0.0241 | 0.4049 |
| 27 | 59 | NDUFA13 | 0.7610 | 0.0698 | 0.7442 |
| 28 | 59 | NDUFA3 | 0.7610 | 0.0299 | 0.4741 |
| 29 | 59 | NDUFB2 | 0.7610 | 0.0767 | 0.7243 |
| 30 | 58 | NDUFB6 | 0.7185 | 0.0285 | 0.5451 |
| 31 | 58 | NDUFS5 | 0.7185 | 0.0501 | 0.5371 |
| 32 | 58 | NDUFA10 | 0.7185 | 0.0662 | 0.6325 |
| 33 | 58 | NDUFV3 | 0.7185 | 0.0641 | 0.6256 |
| 34 | 58 | SRC | 0.7185 | 0.0771 | 0.7579 |
| 35 | 57 | MTOR | 0.6727 | 0.0611 | 0.7397 |
| 36 | 57 | MMP2 | 0.6727 | 0.0426 | 0.6244 |
| 37 | 57 | MYC | 0.6727 | 0.0627 | 0.5951 |
| 38 | 57 | EGFR | 0.6727 | 0.0518 | 0.604 |
| 39 | 57 | TLR4 | 0.6727 | 0.0474 | 0.7191 |
| 40 | 56 | IL4 | 0.6842 | 0.0522 | 0.4733 |
| 41 | 56 | CAT | 0.6842 | 0.0796 | 0.5402 |
| 42 | 56 | RELA | 0.6842 | 0.0224 | 0.7297 |
| 43 | 56 | NDUFB8 | 0.6842 | 0.0249 | 0.7983 |
| 44 | 56 | NDUFA9 | 0.6842 | 0.0396 | 0.5992 |
| 45 | 55 | NDUFB10 | 0.6537 | 0.0283 | 0.7642 |
| 46 | 55 | NDUFA6 | 0.6537 | 0.0384 | 0.4194 |
| 47 | 55 | NDUFB5 | 0.6537 | 0.0161 | 0.6042 |
| 48 | 54 | NDUFS3 | 0.6537 | 0.0435 | 0.4264 |
| 49 | 54 | NDUFS4 | 0.6537 | 0.0789 | 0.7511 |
| 50 | 54 | NDUFA4 | 0.6537 | 0.0233 | 0.4253 |
| 51 | 54 | NDUFA8 | 0.6537 | 0.0555 | 0.6428 |
| 52 | 54 | NDUFA5 | 0.6537 | 0.0605 | 0.4679 |
| 53 | 54 | NDUFA1 | 0.6537 | 0.0313 | 0.7367 |
| 54 | 54 | NDUFB4 | 0.6537 | 0.0461 | 0.5598 |
| 55 | 54 | NDUFB7 | 0.6537 | 0.074 | 0.7374 |
| 56 | 54 | NDUFS7 | 0.6537 | 0.0304 | 0.6477 |
| 57 | 53 | NDUFB3 | 0.6644 | 0.0439 | 0.7098 |
| 58 | 53 | NDUFA2 | 0.6644 | 0.0655 | 0.6076 |
| 59 | 53 | NDUFS6 | 0.6644 | 0.0104 | 0.583 |
| 60 | 53 | NDUFB11 | 0.6644 | 0.0635 | 0.6423 |
| 61 | 53 | NDUFB9 | 0.6644 | 0.0612 | 0.7267 |
| 62 | 53 | NDUFC2 | 0.6644 | 0.0797 | 0.6689 |
| 63 | 53 | NDUFS8 | 0.6644 | 0.0705 | 0.541 |
| 64 | 53 | NDUFV1 | 0.6644 | 0.0379 | 0.6492 |
| 65 | 53 | NDUFV2 | 0.6644 | 0.0292 | 0.5839 |
| 66 | 53 | NDUFS2 | 0.6644 | 0.0444 | 0.7128 |
| 67 | 52 | HMOX1 | 0.6219 | 0.0434 | 0.6914 |
| 68 | 52 | MCL1 | 0.6219 | 0.0186 | 0.5676 |
| 69 | 52 | APP | 0.6219 | 0.0621 | 0.7549 |
| 70 | 52 | JAK2 | 0.6219 | 0.0717 | 0.4264 |
| 71 | 50 | IFNG | 0.6579 | 0.0596 | 0.659 |
| 72 | 50 | EP300 | 0.6579 | 0.0255 | 0.5029 |
| 73 | 50 | MPO | 0.6579 | 0.0587 | 0.7868 |
| 74 | 50 | MMP3 | 0.6579 | 0.0173 | 0.4412 |
| 75 | 50 | CCNB1 | 0.6579 | 0.0664 | 0.5823 |
| 76 | 50 | NFE2L2 | 0.6579 | 0.0338 | 0.5338 |
| 77 | 50 | NOS2 | 0.6579 | 0.0363 | 0.7554 |
| 78 | 49 | ADIPOQ | 0.6446 | 0.0425 | 0.5929 |
| 79 | 49 | NOX4 | 0.6446 | 0.0565 | 0.6814 |
| 80 | 48 | SOD1 | 0.6601 | 0.0527 | 0.7621 |
| 81 | 48 | MAPK9 | 0.6601 | 0.0424 | 0.7384 |
| 82 | 48 | NQO1 | 0.6601 | 0.039 | 0.5759 |
| 83 | 48 | GSR | 0.6601 | 0.065 | 0.408 |
| 84 | 48 | MET | 0.6601 | 0.0254 | 0.514 |
| 85 | 47 | HSPB1 | 0.6528 | 0.0789 | 0.5674 |
| 86 | 47 | CCNA2 | 0.6528 | 0.0545 | 0.5378 |
| 87 | 46 | AKT2 | 0.6427 | 0.0089 | 0.5018 |
| 88 | 46 | NCF1 | 0.6427 | 0.0707 | 0.5965 |
| 89 | 46 | HDAC2 | 0.6427 | 0.0133 | 0.5638 |
| 90 | 46 | CDK2 | 0.6427 | 0.0649 | 0.5176 |
| 91 | 45 | IGF2 | 0.6783 | 0.0104 | 0.5049 |
| 92 | 45 | PIK3CA | 0.6783 | 0.0154 | 0.727 |
| 93 | 45 | ALOX5 | 0.6783 | 0.0227 | 0.5527 |
| 94 | 45 | GSK3B | 0.6783 | 0.0457 | 0.7291 |
| 95 | 45 | PRKCD | 0.6783 | 0.0497 | 0.626 |
| 96 | 44 | MMP14 | 0.6104 | 0.009 | 0.5416 |
| 97 | 44 | GSTP1 | 0.6104 | 0.0726 | 0.5771 |
| 98 | 44 | CDK1 | 0.6104 | 0.0761 | 0.6548 |
| 99 | 43 | ABL1 | 0.6297 | 0.0294 | 0.5355 |
| 100 | 43 | CYP19A1 | 0.6297 | 0.0193 | 0.7228 |
| 101 | 43 | MAPT | 0.6297 | 0.0251 | 0.4834 |
| 102 | 42 | HDAC4 | 0.6014 | 0.0627 | 0.4305 |
| 103 | 42 | BACE1 | 0.6014 | 0.0255 | 0.5606 |
| 104 | 41 | PPARA | 0.5729 | 0.0491 | 0.6172 |
| 105 | 40 | IDO1 | 0.5893 | 0.0677 | 0.6542 |
| 106 | 40 | AKR1B1 | 0.5893 | 0.0217 | 0.7347 |
| 107 | 40 | HSF1 | 0.5893 | 0.024 | 0.5351 |
| 108 | 39 | CDK5 | 0.5801 | 0.0299 | 0.4643 |
| 109 | 39 | CAPN2 | 0.5801 | 0.0266 | 0.6728 |
| 110 | 38 | BCL2 | 0.5774 | 0.0329 | 0.7789 |
| 111 | 37 | PTK2B | 0.5825 | 0.0713 | 0.7104 |
| 112 | 36 | PRKCB | 0.5743 | 0.0216 | 0.4199 |
| 113 | 35 | SPHK1 | 0.5692 | 0.013 | 0.4133 |
| 114 | 35 | CALM1 | 0.5692 | 0.009 | 0.4729 |
| 115 | 35 | PDGFRA | 0.5692 | 0.0726 | 0.7128 |
| 116 | 34 | LRRK2 | 0.5345 | 0.0761 | 0.4123 |
| 117 | 33 | CHUK | 0.5732 | 0.0294 | 0.6217 |
| 118 | 32 | GCLC | 0.5594 | 0.0193 | 0.5972 |
| 119 | 32 | G6PD | 0.5594 | 0.0251 | 0.6095 |
| 120 | 32 | APEX1 | 0.5594 | 0.0627 | 0.5087 |
| 121 | 31 | P4HB | 0.5605 | 0.0255 | 0.7091 |
| 122 | 30 | CNR1 | 0.5429 | 0.0491 | 0.7222 |
| 123 | 30 | CHRNA4 | 0.5429 | 0.0677 | 0.4569 |
| 124 | 29 | DRD2 | 0.5397 | 0.0217 | 0.4438 |
| 125 | 29 | DRD4 | 0.5397 | 0.024 | 0.4949 |
| 126 | 29 | DRD3 | 0.5397 | 0.0299 | 0.6907 |
| 127 | 28 | ADRA2A | 0.5474 | 0.0266 | 0.6455 |
| 128 | 27 | COMT | 0.5336 | 0.0329 | 0.4527 |
| 129 | 27 | COL1A1 | 0.5336 | 0.0713 | 0.6505 |
| 130 | 26 | MAOB | 0.5594 | 0.0216 | 0.7871 |
| 131 | 26 | MAOA | 0.5594 | 0.013 | 0.6305 |
| 132 | 26 | SLC6A3 | 0.5594 | 0.0529 | 0.7537 |
| 133 | 25 | SLC6A4 | 0.5305 | 0.0103 | 0.4323 |
| 134 | 25 | PTGS1 | 0.5305 | 0.0762 | 0.5048 |
| 135 | 25 | INSR | 0.5305 | 0.0682 | 0.4893 |
| 136 | 24 | BAD | 0.5291 | 0.0418 | 0.5431 |
| 137 | 24 | NR1H4 | 0.5291 | 0.0459 | 0.5813 |
| 138 | 23 | HRH3 | 0.5127 | 0.0736 | 0.7931 |
| 139 | 22 | MDH1 | 0.5073 | 0.0179 | 0.4018 |
| 140 | 22 | SLC6A2 | 0.5073 | 0.0587 | 0.544 |
| 141 | 22 | ADORA2A | 0.5073 | 0.0475 | 0.4153 |
| 142 | 21 | XDH | 0.4986 | 0.0622 | 0.598 |
| 143 | 20 | GRM4 | 0.4887 | 0.0665 | 0.5376 |
| 144 | 19 | VCP | 0.4928 | 0.0494 | 0.4052 |
| 145 | 19 | CAMK2A | 0.4928 | 0.0635 | 0.7328 |
| 146 | 18 | HTR2A | 0.4836 | 0.044 | 0.6089 |
| 147 | 18 | DRD1 | 0.4836 | 0.0682 | 0.4232 |
| 148 | 18 | ADRA1A | 0.4836 | 0.0437 | 0.5093 |
| 149 | 17 | PDK1 | 0.5126 | 0.0632 | 0.596 |
| 150 | 17 | PCNA | 0.5126 | 0.0588 | 0.5496 |
| 151 | 16 | ADH1B | 0.4993 | 0.0417 | 0.6916 |
| 152 | 16 | ADH1C | 0.4993 | 0.0458 | 0.7666 |
| 153 | 15 | ADH1A | 0.4746 | 0.0103 | 0.4978 |
| 154 | 15 | ALDH2 | 0.4746 | 0.0204 | 0.757 |
| 155 | 14 | GLO1 | 0.4978 | 0.0557 | 0.4423 |
| 156 | 13 | EGLN1 | 0.4721 | 0.0466 | 0.5034 |
| 157 | 12 | ALDH1A1 | 0.4832 | 0.033 | 0.62 |
| 158 | 11 | ERN1 | 0.4964 | 0.0347 | 0.7712 |
| 159 | 11 | GOT1 | 0.4964 | 0.0257 | 0.7985 |
| 160 | 11 | ARG2 | 0.4964 | 0.0127 | 0.7923 |
| 161 | 11 | DAPK1 | 0.4964 | 0.0562 | 0.4977 |
| 162 | 10 | GABRA2 | 0.4653 | 0.0498 | 0.655 |
| 163 | 10 | IL6ST | 0.4653 | 0.0343 | 0.7328 |
| 164 | 10 | TRAP1 | 0.4653 | 0.0335 | 0.6089 |
| 165 | 9 | P2RY1 | 0.4785 | 0.0275 | 0.4232 |
| 166 | 9 | AVPR1A | 0.4785 | 0.0142 | 0.5093 |
| 167 | 9 | GYS1 | 0.4785 | 0.029 | 0.596 |
| 168 | 9 | CYP1A2 | 0.4785 | 0.0749 | 0.5496 |
| 169 | 9 | ADA | 0.4785 | 0.0143 | 0.6916 |
| 170 | 9 | AKR1C1 | 0.4785 | 0.0275 | 0.5016 |
| 171 | 8 | PPARD | 0.4639 | 0.0136 | 0.4433 |
| 172 | 8 | ADH4 | 0.4639 | 0.0182 | 0.5828 |
| 173 | 8 | CHRNA3 | 0.4639 | 0.0165 | 0.546 |
| 174 | 8 | PYGL | 0.4639 | 0.016 | 0.6103 |
| 175 | 8 | ESRRB | 0.4639 | 0.0741 | 0.4894 |
| 176 | 7 | SLC18A3 | 0.4840 | 0.05 | 0.6114 |
| 177 | 7 | SLC22A3 | 0.4840 | 0.0209 | 0.5758 |
| 178 | 6 | ABAT | 0.4517 | 0.074 | 0.7425 |
| 179 | 6 | KMO | 0.4517 | 0.0145 | 0.6179 |
| 180 | 5 | QDPR | 0.4671 | 0.0318 | 0.5927 |
| 181 | 5 | ABCC2 | 0.4671 | 0.0747 | 0.4259 |
| 182 | 4 | AADAT | 0.4594 | 0.0111 | 0.6841 |
| 183 | 4 | CYP11B2 | 0.4594 | 0.066 | 0.6143 |
| 184 | 4 | SLC1A1 | 0.4594 | 0.0373 | 0.6199 |
| 185 | 3 | SLC29A1 | 0.4486 | 0.078 | 0.5012 |
| 186 | 3 | CYP11B1 | 0.4486 | 0.0793 | 0.5356 |
| 187 | 3 | GRIK5 | 0.4486 | 0.0691 | 0.7396 |
| 188 | 3 | HKDC1 | 0.4486 | 0.0576 | 0.5016 |
| 189 | 3 | SIGMAR1 | 0.4486 | 0.0359 | 0.4433 |
| 190 | 2 | GFPT1 | 0.4602 | 0.0118 | 0.5828 |
| 191 | 1 | AKR1C4 | 0.4571 | 0.0577 | 0.546 |
| 192 | 1 | EIF6 | 0.4571 | 0.0577 | 0.6103 |
