# Supplementary material for: Dihuang-Yinzi Alleviates Cognition Deficits via Targeting Energy-Related Metabolism in an Alzheimer Mouse Model as Demonstrated by Integration of Metabolomics and Network Pharmacology
Source: Front Aging Neurosci. 2022 Apr 1;14:873929. doi: 10.3389/fnagi.2022.873929 (PMC9011333; doi:10.3389/fnagi.2022.873929)
Supplement: Supplementary file 4 [file Table_4.DOCX]

**Supplementary TABLE 4** GO enrichment analysis of targetsof DHYZ against AD

| GO term | subgroup | Enrichment Score |
| --- | --- | --- |
| generation of precursor metabolites and energy | Biological Processes | 1E-100 |
| response to oxidative stress | Biological Processes | 7.26335E-95 |
| cellular response to chemical stress | Biological Processes | 4.15503E-74 |
| energy derivation by oxidation of organic compounds | Biological Processes | 6.99831E-74 |
| cellular response to oxidative stress | Biological Processes | 1.30416E-71 |
| response to inorganic substance | Biological Processes | 3.21917E-70 |
| response to reactive oxygen species | Biological Processes | 3.40245E-67 |
| electron transport chain | Biological Processes | 7.00029E-64 |
| ATP metabolic process | Biological Processes | 3.64003E-62 |
| mitochondrial electron transport, NADH to ubiquinone | Biological Processes | 7.5446E-61 |
| mitochondrial respiratory chain complex I | Cellular Components | 4.53568E-63 |
| NADH dehydrogenase complex | Cellular Components | 4.53568E-63 |
| respiratory chain complex I | Cellular Components | 4.53568E-63 |
| oxidoreductase complex | Cellular Components | 4.91519E-55 |
| respiratory chain complex | Cellular Components | 1.25623E-51 |
| mitochondrial respirasome | Cellular Components | 3.27662E-51 |
| respirasome | Cellular Components | 1.35064E-48 |
| inner mitochondrial membrane protein complex | Cellular Components | 3.83426E-43 |
| mitochondrial envelope | Cellular Components | 6.98848E-39 |
| mitochondrial membrane | Cellular Components | 8.91521E-37 |
| oxidoreductase activity | Molecular Functions | 1.93989E-65 |
| oxidoreductase activity, acting on NAD(P)H, quinone or similar compound as acceptor | Molecular Functions | 9.98134E-64 |
| electron transfer activity | Molecular Functions | 3.4677E-62 |
| NAD(P)H dehydrogenase (quinone) activity | Molecular Functions | 1.79067E-61 |
| oxidoreductase activity, acting on NAD(P)H | Molecular Functions | 7.74263E-61 |
| NADH dehydrogenase activity | Molecular Functions | 2.12479E-60 |
| NADH dehydrogenase (ubiquinone) activity | Molecular Functions | 2.12479E-60 |
| NADH dehydrogenase (quinone) activity | Molecular Functions | 2.12479E-60 |
| protein kinase binding | Molecular Functions | 2.67446E-18 |
| kinase binding | Molecular Functions | 9.43292E-18 |
