# Supplementary material for: Dihuang-Yinzi Alleviates Cognition Deficits via Targeting Energy-Related Metabolism in an Alzheimer Mouse Model as Demonstrated by Integration of Metabolomics and Network Pharmacology
Source: Front Aging Neurosci. 2022 Apr 1;14:873929. doi: 10.3389/fnagi.2022.873929 (PMC9011333; doi:10.3389/fnagi.2022.873929)
Supplement: Supplementary file 5 [file Table_5.DOCX]

**Supplementary TABLE 5** KEGG enrichment analysis of targets of DHYZ against AD

| pathway | enrichment | pvalue | count |
| --- | --- | --- | --- |
| Non-alcoholic fatty liver disease (NAFLD) | -68.200691 | 6.2995E-69 | 50 |
| Alzheimer's disease | -62.2429103 | 5.716E-63 | 47 |
| Retrograde endocannabinoid signaling | -53.6087181 | 2.462E-54 | 42 |
| Parkinson's disease | -53.2405325 | 5.7473E-54 | 40 |
| Huntington's disease | -45.5979191 | 2.524E-46 | 39 |
| Oxidative phosphorylation | -43.5009983 | 3.155E-44 | 34 |
| HIF-1 signaling pathway | -29.5786072 | 2.6387E-30 | 25 |
| Pathways in cancer | -29.1570882 | 6.9649E-30 | 36 |
| Hepatitis B | -27.9392268 | 1.1502E-28 | 27 |
| Fluid shear stress and atherosclerosis | -22.9475711 | 1.1283E-23 | 22 |
| AGE-RAGE signaling pathway in diabetic complications | -21.0468444 | 8.9775E-22 | 19 |
| Measles | -20.4703666 | 3.3856E-21 | 21 |
| Central carbon metabolism in cancer | -20.2530373 | 5.5842E-21 | 16 |
| Tuberculosis | -19.9896097 | 1.0242E-20 | 22 |
